# Potential for *Bacillus thuringiensis* and Other Bacterial Toxins as Biological Control Agents to Combat Dipteran Pests of Medical and Agronomic Importance

**DOI:** 10.3390/toxins12120773

**Published:** 2020-12-05

**Authors:** Daniel Valtierra-de-Luis, Maite Villanueva, Colin Berry, Primitivo Caballero

**Affiliations:** 1 Departamento de Agronomía, Biotecnología y Alimentación, Universidad Pública de Navarra, 31006 Pamplona, Spain; daniel.valtierra@unavarra.es (D.V.-d.-L.); maite.villanueva@unavarra.es (M.V.); 2 Bioinsectis SL, Avda Pamplona 123, Mutilva, 31192 Navarra, Spain; 3 Cardiff School of Biosciences, Cardiff University, Cardiff CF10 3AX, UK; berry@cardiff.ac.uk; 4 Institute for Multidisciplinary Research in Applied Biology-IMAB, Universidad Pública de Navarra, Mutilva, 31192 Navarra, Spain

**Keywords:** dipteran pests, *Bacillus thuringiensis*, insecticidal activity, mosquito control, disease vectors, biological control, agronomic importance

## Abstract

The control of dipteran pests is highly relevant to humans due to their involvement in the transmission of serious diseases including malaria, dengue fever, Chikungunya, yellow fever, zika, and filariasis; as well as their agronomic impact on numerous crops. Many bacteria are able to produce proteins that are active against insect species. These bacteria include *Bacillus thuringiensis*, the most widely-studied pesticidal bacterium, which synthesizes proteins that accumulate in crystals with insecticidal properties and which has been widely used in the biological control of insects from different orders, including Lepidoptera, Coleoptera, and Diptera. In this review, we summarize all the bacterial proteins, from *B. thuringiensis* and other entomopathogenic bacteria, which have described insecticidal activity against dipteran pests, including species of medical and agronomic importance.

## 1. Introduction

*Bacillus thuringiensis* (Bt) has been isolated from the most diverse habitats of our planet [1,2], since its discovery, in 1901, and correct scientific description, in 1915. This has led to the characterization of a large number of Bt strains that, as a whole, have revealed an enormous genetic diversity of this bacterium. This genetic diversity corresponds in good measure to the multiple functions that this bacterium plays in natural and transformed ecosystems (agricultural and forestry). Some of the most relevant functions attributed to Bt from the applied point of view are: plant growth-promoting activities, bioremediation of different heavy metals and other pollutants, biosynthesis of metal nanoparticles, production of polyhydroxy alkanoate biopolymer, and anticancer activities [3]. In agriculture it is, without a doubt, the most widely-used bacterium because of its usefulness as a biological pest control agent and as the most important source of insecticidal genes for the construction of resistant transgenic plants (also known as Bt plants) to some of the most important agricultural and forestry pests [4]. Bt is also an efficient biological control agent for insect vectors (mainly mosquitoes) of diseases of importance in the fields of both human and veterinary health [5].

In this review, we give an overview of dipteran-active pesticidal proteins from a range of bacteria with notes on the major source bacteria, and the activity of individual proteins. The nomenclature of these proteins has undergone a recent revision in order to rationalize the proteins into families based on their structures [6]. In this review we will refer to the proteins by their revised names, with reference to their previous designations also given (Table 1 and text).

## 2. The Entomopathogenic Bacterium *Bacillus thuringiensis*

Bt is a Gram-positive, rod-shaped bacterium with the capacity to form resistant spores, classified in the family Bacillaceae [7]. Bt cells, while sporulating, characteristically form a parasporal crystal composed of proteins which are active against a number of insect species from different orders such as Lepidoptera, Diptera, Coleoptera, Hymenoptera, Hemiptera, Orthoptera, as well as other organisms such as mites [7] and nematodes [8]. When a Bt crystal reaches the insect’s gut, it is solubilized to release one or more protoxins. These protoxins are then proteolyzed and activated by midgut proteases and the toxins can bind to and disrupt cell membranes. The binding and insertion of toxins in the membrane triggers the formation of pores and, consequently, the death of the insect [9,10]. The crystal or inclusion body, which is formed by a combination of delta-endotoxins (historically designated as Cry and/or Cyt proteins, with Cry toxins now divided into structural groups in the new nomenclature), can exhibit different forms (bipyramidal, spherical, etc.) and sizes (smaller, equal to, or greater than the size of the spore), which are usually characteristic for each wild Bt strain [7]. The genes that code for these proteins are usually located in native mega plasmids (>100 kb) [11]. The size and number of plasmids harbouring these genes is highly variable for each strain and some of them (the conjugative plasmids) can be transferred from one Bt strain to another [12]. The synthesis of the crystal entails a huge metabolic investment from the cell [13]. Moreover, the high protein expression levels that occur in the stationary growth phase are controlled at the transcriptional, post-transcriptional, and post-translational levels [12]. 

Based on its molecular structure and its homology, the largest group of crystal proteins is formed by the 3-domain Cry proteins. Domain I consists of a bundle of seven antiparallel α-helices and is the pore-forming domain. Domain II consists of three antiparallel β-sheets (a β-prism structure) and is involved in toxin-receptor interactions. Domain III consists of two twisted, antiparallel β-sheets forming a β-sandwich and has roles in receptor binding and pore formation [14,15]. Three-domain Cry toxins are divided into two main types, those with large protoxin forms of ~120–140 kDa and those with smaller 65–70 kDa protoxins that lack the C-terminal region seen in the larger forms. The larval midgut proteases convert these protoxins into an active fragment through proteolytic processing [16,17]. Although this group of Cry proteins share a remarkably similar and conserved three-domain structure, they vary significantly in their amino acid sequences [9,14,18,19]. There are many other proteins previously designated as Cry proteins that do not have the three-domain structure and these include: Etx_Mtx2 proteins, Toxin_10 proteins, and alpha helical toxins [20]. 

In contrast to Cry proteins, Cyt proteins exhibit a general cytolytic (haemolytic) activity in vitro and dipteran specificity in vivo [14,21]. Their three-dimensional structure shows that Cyt proteins are formed by a single domain with a β-sheet surrounded by two α-helical layers [22,23].

## 3. Importance of Dipteran Control

The larvae of dipteran flies can cause serious damage in agriculture. Adults need to feed on liquid food since they do not have the ability to chew but have licking or sucking mouthparts. However, their larvae do have jaws and are able to feed on solid materials. Today there are more than 150,000 species of dipterans described. Among them there are a number of species that have great economic importance for agriculture by affecting numerous crops. Such insects include species of the Tephritidae, Uidiidae, Agromyzidae, Anthomyiidae, and Drosophilidae. Damage to crops is produced by adults when they feed or when they lay eggs because they produce holes in the leaves/fruits, but especially by the larvae because, e.g., when feeding on the foliar parenchyma, they can make galleries that later necrotize. This damage reduces the photosynthetic capacity of the plant. In the Tephritidae family, commonly known as fruit flies, the females lay eggs inside ripe fruits, flowers, leaves or stems, where the larvae will develop. All the members of this family are phytophagous, varying their larval feeding substrates according to the species, among flowers, fruits, seeds, buds, and other plant organs. However, adults feed on sugars and proteins obtained from wild yeast, secretions from Homoptera, and other environmental substances [24]. The Drosophilidae family includes over 3000 described species that are distributed all over the world [25]. Drosophilids are usually abundant in all situations where rotting fruits or vegetable matter is found. Direct damage occurs on fruits when females select ripe fruits or fruits that change colour, to lay eggs. The insect produces a small hole in the fruit surface that triggers a necrotic reaction around itself, manifesting in the form of a yellow stain in the case of citrus fruits. The wound can then be infected by microorganisms (fungi, bacteria, etc.) that cause fruit rots. The larvae feed on the pulp, forming galleries, causing softening and discolouration of the fruit, indicating the start of putrefaction. Finally, these circumstances cause a series of reactions that promote oxidation processes and premature ripening, leading to fruit fall [26]. 

Individuals of species within the dipteran order are usually controlled by their own natural enemies, so that although they may appear in crops, they can exist at low levels that may not be of economic importance. However, when they appear in high numbers in the larval stage and, in the case of Agromyzidae (leaf miners), when this coincides with the seeding period of a crop, they can become pests with very severe effects. In the case of Tephritids and Drosophilids, the greatest damage to the crop occurs when the fruits are ripe, often precluding the spraying with phytosanitary products due to the proximity of the harvest of the fruits. In addition, if there is any condition that reduces the predators or natural parasitoids of the dipteran pest, there is an increase in its prevalence, and this can result in population explosions that are repeated cyclically. For instance, in certain areas, due to the indiscriminate use of pesticides that has interrupted the natural control by parasitoids, some species of miner insects (such as *Liriomyza sativae* (Agromyzidae)) that were not previously considered as pests, are now threatening crops [27,28]. Another example of a dipteran pest causing economically relevant damage to agricultural production is *Ceratitis capitata* (Tephritidae), which is a polyphagous fly affecting more than 250 species of fruits and vegetables [29]. *C. capitata* can survive across a wide range of hosts and climatic conditions and has become established in the Mediterranean region, Africa, the Middle East, Latin America, and Western Australia [30].

Besides the agricultural importance of dipteran pests, mosquitoes, and other biting flies transmit deadly diseases that seriously threaten human health. Approximately 30 of the 400 known mosquito species are able to transmit pathogens to humans. The three most important genera of mosquito vectors are *Anopheles* spp. from the Anophelinae subfamily and *Culex* spp. and *Aedes* spp. from the Culicidae subfamily. Maladies vectored by these mosquitoes include malaria, filariasis, dengue fever, yellow fever, Chikungunya, Zika, and West Nile Virus. Malaria represents the most significant transmissible health threat to humans with an estimated 214 million people worldwide infected in 2015, of whom 438,000 are estimated to have died [31] (http://www.who.int/malaria). From 1999 to 2013, the West Nile Virus was stated to infect 39,557 people in the United States with 1668 deaths reported and many others becoming severely ill [32,33].

## 4. Dipteran-Active Insecticidal Bacterial Toxins

**Figure 1:**
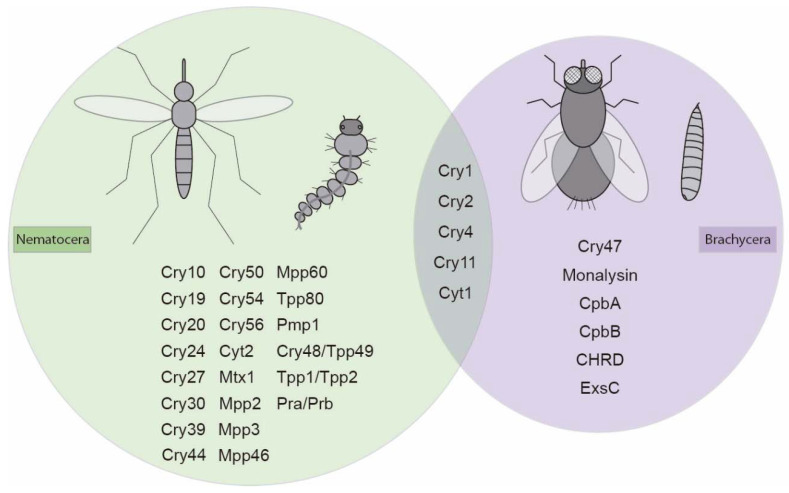
Schematic representation of dipteran active toxins coming from bacteria. Toxins active against the Nematocera suborder are within the green circle. Toxins active against the Brachycera suborder are within the purple circle. Toxins that share activity between suborders are in the overlap between both circles.

A range of Gram-positive and Gram-negative bacteria have reported activities against dipteran insects. In many cases, the content of genes within these bacteria is known and, for some of these, individual toxins have been expressed and assayed to allow us to evaluate their contributions to toxicity. Data on these individual proteins are summarised in Figure 1 and Table 1, and will be discussed in detail below.

### 4.1. Bt Toxins Active against Diptera

Within Bt, there are a number of serovars (including *israelensis*, *jegathesan, darmstadiensis, kyushensis, medellin, fukuokaensis*, *higo*), each comprising a large number of individual Bt strains that contain proteins with known insecticidal activity against an increasing number of dipteran species. An updated list of Bt genes encoding proteins with demonstrated anti-dipteran activity encompasses *cry1, cry2, cry4, cry10, cry11, cry19, cry20, cry24, cry27, cry30, cry39, cry44, cry47, cry50, cry54, cry56, mpp60, tpp80, cyt1,* and *cyt2* (Table 1), and the proteins are described below.

**Table 1:** Summary of LC_50_ values (µg mL^−1^) of pesticidal proteins from Bt and other bacteria against larvae of dipteran species.

Family	Name (Former Name)	Insect Target		Activity Range LC_50_ (µg/mL)	References
		Family	Species		
Cry1	Cry1Ab7	Culicidae	*Aedes aegypti*	ND ^a^	[34]
	Cry1Ac8	Glossinidae	*Glossina morsitans*	0.42–0.74 ^a^	[35]
	Cry1Ba1	Muscidae	*Musca domestica*	20 ^a^	[36]
		Calliphoridae	*Lucilia cuprina*	ND ^a,b^	[37,38]
	Cry1Bc1	Muscidae	*Musca domestica*	79.4 ^a^	[37]
		Calliphoridae	*Lucilia cuprina*	308 ^a^	[37]
			*Chrysomya albiceps*	807 ^a^	[37]
	Cry1Ca1	Culicidae	*Aedes aegypti*	39.3–141 ^a^	[39,40]
			*Anopheles gambiae*	143–283 ^a^	[39]
			*Culex quinquefasciatus*	126 ^a^	[39]
Cry2	Cry2Aa1	Culicidae	*Aedes aegypti*	37.06–79.46 ^a,b^	[41,42,43,44]
			*Anopheles quadrimaculatus*	0.37 ^a^	[44]
			*Aedes triseriatus (Ochlerotatus triseriatus)*	2.84 ^a^	[44]
			*Culex quinquefasciatus*	0.53 ^a^	[45]
	Cry2Aa2	Culicidae	*Culex quinquefasciatus*	1.63 ^a^	[46]
	Cry2Aa4	Culicidae	*Aedes aegypti*	ND ^a^	[47]
			*Anopheles stephensi*	ND ^a^	[47]
			*Culex quinquefasciatus (Culex fatigans)*	ND ^a^	[47]
	Cry2Aa14	Culicidae	*Culex quinquefasciatus*	0.894 ^a^	[48]
	Cry2Aa	Culicidae	*Anopheles gambiae*	0.11 ^a^	[49]
	Cry2Ab1	Culicidae	*Aedes aegypti*	23.42–35.80 ^a,b^	[6,43,50,51,52]
	Cry2Ab2	Culicidae	*Anopheles gambiae*	0.54 ^a^	[49]
	Cry2Ab25	Tephritidae	*Rhagoletis cerasi*	ND ^b^	[53]
	Cry2Ac11	Culicidae	*Aedes aegypti*		[6]
	Cry2Ag	Culicidae	*Aedes aegypti*	2.54 ^b^	[51]
	Cry2Am1	Culicidae	*Aedes aegypti*		[6]
Cry4	Cry4Aa1	Culicidae	*Aedes aegypti*	0.03–13 ^a,b^	[54,55,56,57,58,59,60,61,62,63]
			*Anopheles gambiae*	1.07–1.17 ^a^	[55]
			*Anopheles stephensi*	0.52–7.4 ^a,b^	[59,60,61,64]
			*Culex pipiens*	0.25–0.97 ^a^	[54,59,60,61,65]
			*Culex quinquefasciatus*	0.05–5.04 ^a,b^	[33,54,55,66]
		Simuliidae	*Simulium* spp.	ND ^b^	[67]
	Cry4Ba1	Chironomidae	*Chironomus tepperi*	0.94 ^b^	[68]
		Culicidae	*Aedes aegypti*	0.12–0.94 ^a,b^	[54,55,57,58,59,61,64,69]
			*Anopheles albimanus*	1.3 ^b^	[70]
			*Anopheles gambiae*	0.79 ^a^	[55]
			*Anopheles quadrimaculatus*	0.25 ^a^	[54]
			*Anopheles stephensi*	0.55–17 ^a,b^	[59,61,64]
			*Culex quinquefasciatus*	24.5 ^a^	[55]
		Culicidae	*Culex pipiens*	ND ^b^	[64]
		Tipulidae	*Tipula oleracea*	ND ^a^	[71]
		Simuliidae	*Simulium* spp.	ND ^b^	[67]
	Cry4Ba2	Culicidae	*Aedes aegypti*	ND ^a^	[72]
	Cry4Cb1	Culicidae	*Aedes aegypti*	0.083 ^b^	[73]
Cry10	Cry10Aa	Culicidae	*Aedes aegypti*	0.3–20.61 ^a,b^	[62,74]
Cry11	Cry11Aa1	Chironomidae	*Chironomus tepperi*	0.56 ^b^	[68]
		Culicidae	*Aedes aegypti*	0.01-1.35 ^a,b^	[41,42,57,58,61,75,76,77,78,79]
			*Anopheles albimanus*	0.9 ^b^	[70]
			*Anopheles stephensi*	0.13-0.45 ^a,b^	[60,75,78,80,81]
			*Anopheles albimanus*	6.759 ^a^	[77]
			*Culex pipiens*	0.009-0.27 ^a^	[75,78]
			*Culex quinquefasciatus*	0.01-0.13 ^a,b^	[45,66,76,77,82,83]
		Tipulidae	*Tipula oleracea*	ND ^b^	[81]
		Simuliidae	*Simulium* spp.	ND ^b^	[67]
	Cry11Ba1	Culicidae	*Aedes aegypti*	0.02–0.03 ^a,b^	[75,77,84]
			*Anopheles albimanus*	0.10 ^a^	[77]
			*Anopheles stephensi*	0.04 ^a^	[75]
			*Culex pipiens*	0.01–0.11 ^a^	[61,65]
			*Culex quinquefasciatus*	0.006–0.02 ^a,b^	[77,83,84]
	Cry11Bb1	Culicidae	*Aedes aegypti*	0.02–0.85 ^a,b^	[76,77,85,86]
			*Anopheles albimanus*	0.17 ^a^	[77]
			*Anopheles stephensi*	0.07 ^a^	[85]
			*Culex pipiens*	0.04 ^a^	[85]
			*Culex quinquefasciatus*	0.01–0.13 ^a,b^	[76,77,86]
Cry16-Cry17-Cbm17.1-Cbm17.2	Cry16-Cry17-Cbm17.1-Cbm17.2	Culicidae	*Aedes aegypti*	ND ^b^	[87]
Cry19	Cry19Aa	Culicidae	*Anopheles stephensi*	1.04 ^a^	[88]
			*Culex pipiens*	0.19 ^a^	[88]
	Cry19B	Culicidae	*Culex pipiens molestus*	5.93 ^a^	[89]
Cry20	Cry20Aa1	Culicidae	*Aedes aegypti*	648 ^b^	[90]
			*Culex quinquefasciatus*	700 ^b^	[90]
Cry24	Cry24Ca1	Culicidae	*Aedes aegypti*	0.48 ^b^	[91]
Cry27	Cry27Aa1	Culicidae	*Anopheles stephensi*	94.3 ^a^	[92]
Cry30	Cry30Fa1	Culicidae	*Aedes aegypti*	15.4 ^a^	[93]
	Cry30Ga1	Culicidae	*Aedes aegypti*	7.10 ^b^	[73]
Cry39	Cry39Aa1	Culicidae	*Anopheles stephensi*	0.75 ^b^	[94]
			*Culex pipiens*	41.94 ^b^	[95]
Cry44	Cry44Aa1	Culicidae	*Aedes aegypti*	0.01 ^a^	[60]
			*Anopheles stephensi*	1.26 ^a^	[60]
			*Culex pipiens*	0.006 ^a^	[60]
Mpp46	Mpp46Ab (Cry46Ab)	Culicidae	*Culex pipiens*	1.02 ^a^	[96]
Cry47	Cry47Aa1	Calliphoridae	*Lucilia cuprina*	ND ^a^	[97,98]
Cry50	Cry50Ba	Culicidae	*Culex quinquefasciatus*	0.07 ^a^	[99]
Cry54	Cry54Aa1	Culicidae	*Aedes aegypti*	9.02 ^a^	[100]
Cry56	Cry56Aa1	Culicidae	*Aedes aegypti*	0.15 ^a^	[101]
Mpp60	Mpp60Aa (Cry60Aa)	Culicidae	*Culex quinquefasciatus*	7.9 ^b^	[102]
	Mpp60Ba (Cry60Ba)	Culicidae	*Culex quinquefasciatus*	5.5 ^b^	[102]
	Mpp60Aa+Mpp60Ba (Cry60Aa+Cry60Ba)	Culicidae	*Culex quinquefasciatus*	2.9 ^b^	[102]
Tpp80	Tpp80Aa1 (Cry80Aa1)	Culicidae	*Culex pipiens pallens*	71.9 ^a^	[103]
Cyt1	Cyt1Aa1	Calliphoridae	*Calliphora stygia*	305 ^a^	[104]
			*Lucilia cuprina*	296 ^a^	[104]
			*Lucilia sericata*	236 ^a^	[104]
		Chironomidae	*Chironomus tepperi*	31 ^b^	[68]
		Culicidae	*Aedes aegypti*	0.15–1.86 ^a,b^	[74,105,106,107,108,109]
			*Anopheles stephensi*	2.7–6.3 ^a^	[105,108]
			*Culex pipiens*	0.6–1.2 ^a^	[105,108]
			*Culex quinquefasciatus*	0.4 ^a^	[108]
		Tephritidae	*Ceratitis capitata*	ND ^a^	[26]
		Tipulidae	*Tipula paludosa*	ND ^a^	[110]
	Cyt1Aa2	Culicidae	*Aedes aegypti*	0.12–1.21 ^a,b^	[58,63,111]
			*Anopheles gambiae*	1–2 ^a,b^	[111]
			*Culex pipiens*	0.5–2 ^a,b^	[111]
	Cyt1Aa4	Culicidae	*Aedes aegypti*	0.06 ^a^	[79]
			*Culex quinquefasciatus*	>10 ^a^	[112]
	Cyt1Ab1	Culicidae	*Aedes aegypti*	32.6–59 ^a,b^	[105,113]
			*Anopheles stephensi*	20 ^a^	[105]
			*Culex pipiens*	5.7 ^a^	[105]
			*Culex quinquefasciatus*	32.9–114.5 ^b^	[113]
	Cyt1Ba1	Agromyzidae	*Liriomyza trifolii*	ND ^a^	[114]
Cyt2	Cyt2Aa1	Culicidae	*Aedes aegypti*	1–4 ^a,b^	[111]
			*Anopheles gambiae*	1–2 ^a,b^	[111]
			*Culex pipiens*	0.5–4 ^a,b^	[111]
	Cyt2Aa2	Culicidae	*Aedes aegypti*	0.35–0.5 ^a^	[69,115]
			*Culex quinquefasciatus*	0.25–0.5 ^a^	[69,115]
	Cyt2Aa3	Chironomidae	*Chironomus tepperi*	36 ^a^	[116]
		Culicidae	*Culex quinquefasciatus*	0.53 ^a^	[116]
	Cyt2Ba1	Culicidae	*Aedes aegypti*	0.28–33 ^a,b^	[62,108,113,117]
			*Anopheles stephensi*	5.5 ^a^	[108]
			*Culex pipiens*	5 ^a^	[108]
			*Culex quinquefasciatus*	1.8–31.5 ^a,b^	[108,113]
	Cyt2Bb1	Culicidae	*Aedes aegypti*	6.8 ^b^	[106]
	Cyt2Bc1	Culicidae	*Aedes aegypti*	7 ^a^	[108]
			*Anopheles stephensi*	11 ^a^	[108]
			*Culex pipiens*	7.3 ^a^	[108]
			*Culex quinquefasciatus*	1.8 ^a^	[108]
Mtx	Mtx1Aa1	Culicidae	*Culex quinquefasciatus*	0.01 ^a^	[118]
			*Aedes aegypti*	0.05 ^a^	[118]
		Chironomidae	*Chironomus riparius*	4.06 ^a^	[119]
Mpp2	Mpp2Aa1 (Mtx2)	Culicidae	*Culex quinquefasciatus*	4.13–107 ^b^	[66]
	Mpp2Aa1 (Mtx2 strain SSII-1)	Culicidae	*Culex quinquefasciatus*	0.93 ^a^	[120]
			*Aedes aegypti*	14.5 ^a^	[120]
	Mpp2Aa2 Mtx2 (31-2)	Culicidae	*Culex quinquefasciatus*	3.90 ^a^	[120]
			*Aedes aegypti*	3.91 ^a^	[120]
Mpp3	Mpp3Aa1 (Mtx3)	Culicidae	*Culex quinquefasciatus*	ND ^a^	[121]
			*Aedes aegypti*	ND ^a^	[121]
Monalysin	Monalysin	Drosophilidae	*Drosophila melanogaster*	ND ^a^	[122]
CpbA	CpbA	Muscidae	*Musca domestica*	ND ^a^	[123]
CpbB	CpbB	Muscidae	*Musca domestica*	ND ^a^	[123]
CHRD	CHRD	Muscidae	*Musca domestica*	ND ^a^	[123]
ExsC	ExsC	Muscidae	*Musca domestica*	ND ^a^	[123]
Pmp1	Pmp1	Culicidae	*Anopheles coluzzii*	ND ^a^	[124]
Two part toxins	Cry48Aa/Tpp49 (Cry49Aa)	Culicidae	*Culex quinquefasciatus*	0.02/0.006 ^a^	[125]
	Tpp1/Tpp2 (BinA/BinB)	Culicidae	*Aedes aegypti*	42 ^a^	[126,127]
			*Aedes atropalpus*	ND ^a^	[126]
			*Anopheles gambiae*	0.36 ^a^	[127]
			*Anopheles stephensi*	0.39 ^a^	[127]
			*Anopheles albimanus*	1 ^a^	[127]
			*Anopheles quadrimaculatus*	4.6 ^a^	[127]
			*Culex pipiens*	0.1 ^a^	[127]
		Culicidae	*Culex quinquefasciatus*	0.013–0.03 ^a,b^	[126,128]
	Pra/Prb (PirA/PirB)	Culicidae	*Aedes aegypti*	ND ^a^	[84,129]
Synergy	Cry1Ca/Cyt1Aa	Culicidae	*Aedes aegypti*	0.61 ^a^	[130]
	Cry2Aa/Cry2Ab	Culicidae	*Aedes aegypti*	51.3 ^b^	[43]
	Cry2Aa/Cry50Ba	Culicidae	*Culex quinquefasciatus*	0.05 ^a^	[99]
	Cry4Aa/Cry4Ba	Culicidae	*Aedes aegypti*	0.05 ^a^	[61]
			*Anopheles stephensi*	0.02 ^a^	[61]
			*Culex pipiens*	0.04 ^a^	[61]
			*Culex quinquefasciatus*	1.49–315 ^b^	[66]
		Simuliidae	*Simulium* spp.	ND ^b^	[67]
					
	Cry4Aa/Cyt1Aa	Culicidae	*Aedes aegypti*	0.07 ^a^	[58]
		Chironomidae	*Chironomus tepperi*	44 ^b^	[68]
		Tipulidae	*Tipula paludosa*	ND ^a^	[110]
	Cry4Aa/Cyt2Ba	Culicidae	*Aedes aegypti*	0.013 ^b^	[62,131]
	Cry4Aa/Cry11Ba	Culicidae	*Culex pipiens*	0.04 ^a^	[65]
	Cry4Aa/Cry46Ab	Culicidae	*Culex pipiens*	0.18 ^a^	[96]
	Cry4A/Mtx1Aa1	Culicidae	*Culex quinquefasciatus*	1.06–2.37 ^b^	[66]
	Cry4A/Mpp2Aa1 (Mtx2)	Culicidae	*Culex quinquefasciatus*	0.27–1.21 ^b^	[66]
	Cry4Ba/Cry11Aa	Culicidae	*Anopheles albimanus*	0.567 ^b^	[70]
		Simuliidae	*Simulium* spp.	ND ^b^	[67]
	Cry4Ba/Cyt1Aa	Culicidae	*Aedes aegypti*	0.62 ^a^	[58]
		Culicidae	*Anopheles albimanus*	0.33–0.77 ^b^	[70]
	Cry4Ba/Cyt2Aa2	Culicidae	*Aedes aegypti*	0.007 ^a^	[69]
			*Culex quinquefasciatus*	0.02 ^a^	[69]
	Cry4B/Mtx1Aa1	Culicidae	*Culex quinquefasciatus*	18.2–29.0 ^b^	[66]
	Cry4B/Mpp2Aa1 (Mtx2)	Culicidae	*Culex quinquefasciatus*	85.7 ^b^	[66]
	Cry10Aa/Cyt1Aa	Culicidae	*Aedes aegypti*	0.03–0.08 ^a,b^	[74]
	Cry10Aa/Cyt2Ba	Culicidae	*Aedes aegypti*	0.004 ^b^	[62]
	Cry11Aa/Cyt1Aa	Culicidae	*Aedes aegypti*	0.01–0.12 ^a^	[58,79]
		Culicidae	*Anopheles albimanus*	0.28–0.37 ^b^	[70]
	Cry11/Mtx1Aa1	Culicidae	*Culex quinquefasciatus*	0.66–3.03 ^b^	[66]
	Cry11/Mpp2Aa1 (Mtx2)		*Culex quinquefasciatus*	0.90 ^b^	[66]
	Cry11Bb/Cry29Aa	Culicidae	*Aedes aegypti*	3.94 ^a^	[85]
			*Anopheles stephensi*	2.13 ^a^	[85]
			*Culex pipiens*	0.73 ^a^	[85]
	Cry11Bb/Cry30Aa	Culicidae	*Aedes aegypti*	16.96 ^a^	[85]
			*Anopheles stephensi*	1.43 ^a^	[85]
			*Culex pipiens*	1.13 ^a^	[85]
	Cry4A/Cry4B/Cry11A	Culicidae	*Aedes aegypti*	0.12 ^a^	[58]
			*Culex quinquefasciatus*	0.008–0.59 ^b^	[66]
	Cry4A/Cry4B/Cyt1A	Culicidae	*Aedes aegypti*	0.08 ^a^	[58]
	Cry4A/Cry4B/Mtx1Aa1	Culicidae	*Culex quinquefasciatus*	0.18–0.77 ^b^	[66]
	Cry4A/Cry4B/Mpp2Aa1 (Mtx2)	Culicidae	*Culex quinquefasciatus*	0.11–0.32 ^b^	[66]
	Cry4A/Cry4B/Cry11A/Cyt1A	Culicidae	*Aedes aegypti*	0.08 ^a^	[58]
			*Culex quinquefasciatus*	0.02–0.07 ^b^	[66]
	Cry4A/Cry4B/Cry11A/Mtx1Aa1	Culicidae	*Culex quinquefasciatus*	0.02–0.24 ^b^	[66]
	Cry4A/Cry4B/Cry11A/Mpp2Aa1 (Mtx2)	Culicidae	*Culex quinquefasciatus*	0.03–0.06 ^b^	[66]
	Cry4A/Cry4B/Cry11A/Cyt1A/Mtx1Aa1	Culicidae	*Culex quinquefasciatus*	0.02–0.06 ^b^	[66]
	Cry4A/Cry4B/Cry11A/Cyt1A/Mpp2Aa1(Mtx2)	Culicidae	*Culex quinquefasciatus*	0.30–1.09 ^b^	[66]
	Cry4Ba/Cry11Aa/Cyt1Aa	Culicidae	*Anopheles albimanus*	0.7–8.33 ^b^	[70]
	Cry11Bb/Cry29Aa/Cry30Aa	Culicidae	*Aedes aegypti*	5.43 ^a^	[85]
			*Anopheles stephensi*	1.31 ^a^	[85]
			*Culex pipiens*	0.85 ^a^	[85]

a. Purified proteins. b. Powders of recombinant Bt containing spore/crystal mixtures. ND, toxicity reported but LC_50_ not determined.

#### 4.1.1. Cry Toxins from Bt ser. *israelensis*

**Figure 2:**
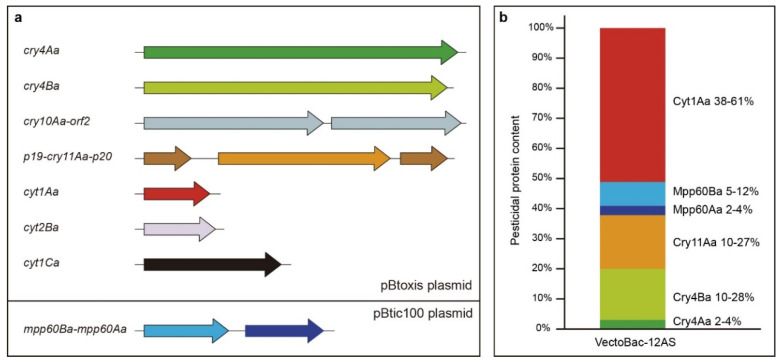
Pesticidal protein composition of Bti strains. (**a**), Scheme of all genes and operons that may be present in Bti strains, which encode crystal proteins. (**b**), Relative molar composition of proteins in the parasporal crystal of Bti-based Vectobac-12AS insecticide. The composition is expressed as a range from two independent tryptic digestions with two technical replicates each (adapted from [139]).

Bt ser. *israelensis* (Bti) was the first Bt serotype found to be toxic against dipteran larvae [132]. Bti is a highly potent and environmentally friendly biological alternative component in integrated programs to control disease vectors [133,134]. Bti was much more effective against many species of mosquito and black fly larvae than any previously known bio-control agent [135]. The Bti crystal is potentially composed of up to six crystal proteins (Cry4Aa, Cry4Ba, Cry10Aa, Cry11Aa, and Mpp60A/Mpp60B (formerly Cry60A/Cry60B)) although strains may lack *mpp60* genes [136] and three Cyt proteins (Cyt1Aa, Cyt2Ba, and Cyt1Ca). Most of the genes encoding these toxins are present in the 128 kb plasmid pBtoxis [136]; however, some Bti strains contain another plasmid named pBtic100, which carries two additional pesticidal protein genes, encoding Mpp60Aa and Mpp60Ba toxins [137,138] (Figure 2a). Bti has been the most studied serovar over the years and most of the Bt products marketed are based on strains in this serovar. Pesticidal proteins from Bti (Cry4A, Cry4B, Cry11A, and Cyt1A) have also been extensively studied; however, the other proteins, which may have lower expression, have been much less studied (Cry10Aa, Cyt2Ba, Mpp60Aa, and Mpp60Ba). Their activity, as well as the possible interactions between them, opens new study possibilities in the search for alternatives for dipteran control.

##### Cry4 Proteins

Bti strains may produce two members of the large protoxin 3-domain Cry family: Cry4Aa (135 kDa) and Cry4Ba (128 kDa). These 3-domain protoxins form crystals spontaneously via inter- and intra-molecular disulphide bonds by their conserved C-terminal halves [140,141]. 

The Cry4Aa target range covers the following mosquito species: *Aedes aegypti*, *Anopheles stephensi, Anopheles gambiae*, *Culex pipiens,* and *Culex quinquefasciatus*. Several studies have provided evidence that the *Culex* species are the most susceptible to these proteins while the species of the *Anopheles* and *Aedes* genera are less susceptible [55,57,59,61,142,143]. 

Cry4Ba is another of the major crystal proteins produced by Bti. This protein showed high toxic activity against *A. aegypti* and *An. stephensi* larvae but it was totally inactive against larvae in the genus *Culex* [54,55,59,61,69]. The toxicity of Cry4Ba toxin toward *A. aegypti* and *An. stephensi* larvae is higher than that of Cry4A [59]. The putative loops 1 and 2 of domain II of the protein are responsible for its activity and mutations in putative loop 3 produce an increase in toxicity against *Culex* [54]. Cry4Ba also has toxicity against *Chironomus tepperi* (Diptera; Chironomidae) [68] and is the most effective Bti toxin against *Simulium* spp. (lower activity seen for Cry4Aa) [67]. 

##### Cry10Aa Protein

The Cry10Aa protein is a minor component of the crystal produced by the Bt strains of ser. *israelensis* [144,145,146] and, unlike Cry4Aa and Cry4Ba, is a short protoxin 3-domain protein. However, the 2025 bp *cry10Aa* gene (*orf1*) is followed (after a 66 nt gap) by a second gene (*orf2*) that encodes a sequence that is similar to the carboxyl end of the Cry4Aa and Cry4Ba proteins [136]. The *orf2* codes for a 56 kDa protein and, therefore, when the complete operon is cloned, two proteins of 68 (*orf1*) and 56 (*orf2*) kDa are expressed [62,74]. Parasporal bodies formed by the complete Cry10Aa (Orf1-Orf2) are as active to *A. aegypti* as the Cry4 toxins [62,74]. 

##### Cry11 Proteins

The Cry11 family also belongs to the large group of δ-endotoxins comprising three structural domains and is composed of proteins active against dipteran targets [61,76,147]. Cry11Aa from Bti is a 72 kDa protoxin that is located in an operon where the main gene (1941 bp) is flanked by two other small genes known as *p19* and *p20* [148,149]. Among individual toxins from Bti, Cry11Aa, together with Cry4Ba, are the second most abundantly produced toxins, after Cyt1Aa (Figure 2b) [139]. Cry11Aa proteins have a high toxicity against both *Aedes* and *Culex* genera while their insecticidal activity is lower against larvae of *Anopheles* species [41,61,80,82,150]. This protein is activated in the insect midgut by proteolytic cleavage resulting in two fragments of 38 and 30 kDa with the capacity to bind the midgut microvilli [57,78,151]. 

In the case of *A. aegypti*, Cry11Aa may interact with different midgut brush border membrane receptors; a GPI anchored alkaline phosphatase (GPI-ALP) [152], an aminopeptidase N [153] and a cadherin [154]. The protein also binds to Cyt1Aa as a membrane-bound receptor, increasing its activity [155]. In *Anopheles albimanus,* an alpha-amylase has been described as a putative binding receptor for Cry11Aa [70]. There are other midgut proteins, such as ATP binding protein, that increase the toxicity of this protein against *C. quinquefasciatus* third instar larvae [33]. Cry11Aa is also toxic against other dipterans such as *Chironomus tepperi* (Diptera; Chironomidae)*, Tipula oleracea* and *Simulium* species [67,68,81].

Two other Cry11 proteins, Cry11Bb (94 kDa) produced by Bt ser. *medellin* and Cry11Ba (81 kDa) produced by Bt ser. *jegathesan,* share similar insect specificity and their activity is higher than that of Cry11Aa [75,77]. Three different *A. aegypti* midgut proteins, cadherin, AaeALP1, and AaeAPN1, are involved in Cry11Ba binding to *A. aegypti* midgut brush border membranes [156].

##### Mpp60A/Mpp60B Proteins (Formerly Cry60A/Cry60B)

In Bt ser. *jegathesan,* the *mpp60A* (960 bp) and *mpp60B* (912 bp) genes form an operon. These two ORFs have also been detected in Bt ser. *malayensis* 4AV1 [102] and Bt ser. *israelensis* ATCC 35646 [157]. Interestingly, the operon that contains the *mpp60A* and *mpp60B* genes has exactly the same structure in these three Bt strains, classified in three different serovars. Both proteins belong to the Etx/Mtx2 protein family. Individual or joint expression of the *mpp60A* and *mpp60B* genes in a Bt strain (acrystalliferous) produces crystal components (33 and 35 kDa, respectively) that show moderate insecticidal activity against fourth instar *C. quinquefasciatus* larvae. Despite being part of the same operon, Mpp60Aa and Mpp60Ba should not be considered binary toxins because neither of them depends on the other to be expressed or to exert its insecticidal activity on the target insect [102].

#### 4.1.2. Other Toxins Specific to Diptera

In addition to the Bt ser. *israelensis* toxins, there are a number of other 3-domain Cry proteins from different Bt serovars with toxicity against several species of Diptera. For example, Cry19Aa, identified in a Bt ser. *jegathesan* strain, together with ORF2 (encoded in a similar operon structure to the *cry10-orf2* format of Bti), exhibited toxic activity against *C. pipiens* and *An. stephensi* [88]. Cry19Ba, a close member of this family derived from Bt ser. *higo*, showed activity against *Culex molestus* larvae, but not against *An. stephensi* [89]. Cry20Aa is another mosquitocidal protein that has been shown to be toxic to larvae of *A. aegypti* and *C. quinquefasciatus* and is produced by a strain of Bt ser. *fukuokaensis*. Nevertheless, the toxicity was not high, perhaps due to the rapid degradation of the protein [90]. Cry24Ca protein also exhibited larvicidal activity against *A. aegypti* [91]. Within the Cry27 family, it has been reported that the Cry27Aa protein produced by a strain of Bt ser. *higo* shows activity against *An. stephensi* but is not toxic to species classified in the genera *Culex* or *Aedes* [92]. The Cry39Aa protein has also been found to be highly toxic against *An. stephensi* larvae [60,95]. In contrast, Cry44Aa from Bt ser. *entomocidus,* showed high toxic activity against *Culex pipiens* and *A. aegypti,* although the activity against *An. stephensi* was lower [60]. The aerolysin-like Mpp46Ab (previously known as Cry46Ab and also designated parasporin-2Ab) and Cry50Ba have been described as highly active against *C. pipiens* and *C. quinquefasciatus* larvae, respectively [65,99]. Tpp80Aa (previously known as Cry80Aa) also showed toxicity against *C. pipiens* [103]. Finally, Cry47Aa, has also been described as active against dipteran species, such as the sheep blowfly *Lucilia cuprina* (Diptera; Calliphoridae) [97,98].

#### 4.1.3. Anti-Dipteran Toxins with Cross-Order Activity

##### Cry1 Protein

The Cry1 family is typically active against species of the lepidopteran order [158]. However, several proteins belonging to this family also display insecticidal activity against species in the Nematocera and Brachycera suborders of Diptera. For example, Cry1Ab7 protein, was active against *A. aegypti* larvae [34] and Cry1Ca showed toxic activity against larvae of different mosquito species such as *A. aegypti*, *C. quinquefasciatus* and *An. gambiae* [39,40]. Other Cry1 proteins show activity against different species of flies. Cry1Ac, for example, was active against *Glossina morsitans* adults (Diptera; Glossinidae) [35] and Cry1Ba had toxicity against *Musca domestica* larvae (Diptera; Muscidae) [36] and also *Lucilia cuprina* larvae (Diptera; Calliphoridae), when it was applied in high concentrations [38].

##### Cry2A Protein

Within the Cry2 family, it has been described that the Cry2A proteins have a wide activity that can include species of the orders Lepidoptera and Diptera [50,159]. Some Cry2Aa variants are toxic against the dipteran order, with activities demonstrated mostly using *A. aegypti* [42,44,47,52,160,161], but targets include *C. quinquefasciatus (Culex fatigans), An. stephensi* and *An gambiae* [44,46,47,49]. Cry2Ab2, was reported to have a much narrower target range with high-level activity against *An. gambiae* but no activity against *A. aegypti* or *C. pipiens* [49]. Cry2Ag has also been reported as active against the larvae of *A. aegypti* [51], while Cry2Ab25 has shown high mortality against *Rhagoletis cerasi* larvae (Diptera; Tephritidae) [53]. An assessment of a number of Cry2A variants and their activity against *A. aegypti* has recently been published [161]. The role of a region at the N-terminus of Cry2 proteins in activity against *A. aegypti* has also been described [162].

##### Other Cry Proteins

There are a number of proteins that are simultaneously active against larvae of several dipteran and lepidopteran species. This is the case for two proteins from the Cry30 family. Both, Cry30Fa and Cry30Ga1, had remarkable insecticidal effects against *A. aegypti* and *Plutella xylostella* [73,93]. In addition, Cry54Aa protein has shown activity against *A. aegypti* larvae, as well as against the Lepidoptera *Spodoptera exigua* (*Laphygma exigua*) and *Helicoverpa armigera* [100]. Finally, Cry56Aa was toxic to both dipteran (*A. aegypti*) and lepidopteran (*P. xylostella* and *H. armigera*) pests [101]. 

For all of the Cry proteins above, with cross-order activity, future work to elucidate the basis of specificity will be of great interest (for instance, whether different regions of the toxins are involved in receptor recognition for different targets) and exploration of their structures, in combination with receptor structures, may yield an increased understanding of their molecular interactions and activity. This, in turn, may allow future expansion of the battery of toxins available to combat dipteran pests.

#### 4.1.4. Anti-Dipteran Cyt Toxins

##### Cyt1 Proteins

The proteins of the Cyt1 family do not bear any similarity with any of the Cry families currently described (including those recently renamed into other structural classes) [6]. Of all the proteins described in the Cyt1 family, the Cyt1Aa protein has undoubtedly been the most widely studied. Cyt1Aa is the main component of Bti crystals (Figure 2b) and it adopts a typical cytolysin fold containing a β-sheet held by two surrounding alpha-helical layers [22]. The insecticidal activity of Cyt1Aa for the larvae of various dipteran species has been reported by several authors [71,79,143,163]. Efficient expression of the Cyt1Aa (molecular mass of 27 kDa) protein requires the presence of a 20 kDa “helper” polypeptide [164]. Proteolytic digestion of Cyt1Aa protein produces fragments of 22–25 kDa that are more effective than the native protoxin in vitro [165]. This toxin shows haemolytic and cytolytic in vitro activity to cells of vertebrates and invertebrates [63,142,166], apparently due to the interaction between its hydrophobic segment and membrane phospholipids from the midgut epithelial cells [167,168,169,170]. However, differences in activity against insects and red blood cells have been described [171]. Cyt1Aa has been tested as a full-length solubilised protein, mixed with diet, against a number of species of the Brachycera suborder and has been found to be toxic against first-instar *Lucilia sericata* (Diptera; Calliphoridae), *Lucilia cuprina* (Diptera; Calliphoridae) and *Calliphora stygia* (Diptera; Calliphoridae) [104]. In these experiments, trypsin treatment of the solubilised toxin increased activity 4–6-fold but non solubilised purified Cyt1Aa crystals were not toxic. Cyt1Aa has also shown toxic activity against *Tipula paludosa* larvae [110]. 

To date, no highly toxic Bt toxins have been found against *Ceratitis capitata* (Diptera; Tephritidae) in the field. However, some authors showed that, under controlled laboratory conditions, solubilized Cyt1A protoxin showed moderate toxicity against *C. capitata* larvae [26]. Bti crystals do not appear to be solubilized efficiently below pH 9 and the pH of *C. capitata* third instar larvae and adult midgut has been calculated as 8 and 7.5, respectively [172]. The development of more accurate and reproducible quantitative methods may help in the determination of toxic properties for insecticidal pathogens that act by ingestion in adult Diptera [173].

Cyt1Ab, a protein that shares 86% identity with Cyt1Aa, is also active, although to a lesser extent than Cyt1Aa, against *Aedes*, *Anopheles,* and *Culex* larvae [105]. The ability of Cyt1Ba to induce mortality and reduce the damage caused by *Liriomyza trifolii* (Diptera; Agromyzidae) mining larvae has also been described [114]. Cyt1Ca is approximately twice the size of the other Cyt proteins and, in addition to the Cyt-like region, it has an extra C-terminal lectin-like domain. No activity or haemolytic effect has been observed for this protein encoded in the pBtoxis plasmid of Bti [131].

##### Cyt2 Proteins

Proteins of the Cyt2 family have been identified and characterized in several Bt serovars: Cyt2Aa from Bt ser. *kyushensis* [111] and *darmstadiensis* [115], Cyt2Ba from Bti [174], Cyt2Bb from Bt ser. *jegathesan* [106] and Cyt2Bc from Bt ser. *medellin* [108]. 

Cyt2Aa1 from Bt ser. *kyushensis* displays low identity (39%) with Cyt1Aa from Bti, but the similarity is 70% [174]. Moreover, both are processed in similar domains [111] probably because they share a high degree of structural similarity [111,175]. Cyt2Aa1 is a 29.2 kDa protein and its crystal structure has been solved [175]. It consists of a single α-β domain comprising two outer layers of α-helix hairpins and a β-sheet in between. The protein does not show haemolytic activity as a protoxin; however, N- and C- terminal segments are cleaved by proteolysis leading to dimer dissociation and toxin activation. Cyt2Aa1 showed LC_50_ values in a range of 0.5 and 4 µg/mL against *Culex*, *Anopheles,* and *Aedes* larvae [111]. Cyt2Aa2 produced by Bt ser. *darmstadiensis* exhibited moderate activity against *Culex* and *Aedes* larvae and haemolytic activity against sheep erythrocytes [115]. Cyt2Aa3, from Bt strain MC28, exhibited toxic activity against *Ch. tepperi* (Diptera; Chironomidae) and *C. quinquefasciatus* larvae [116].

The Cyt2Ba1 protein (30.1 kDa) from Bti shows a 41% identity with Cyt1Aa1 and 67% with Cyt2Aa1 [174]. It was less active than Cyt1Aa against *A. aegypti*, *C. pipiens*, *C. quinquefasciatus* and *An. stephensi* larvae [108,113]. Moreover, solubilisation or trypsin activation was essential for its haemolytic activity [108]. The crystal structure of the proteolytically cleaved active form of Cyt2Ba has been described [23] and resembles that of the protoxin form of Cyt2Aa and also the fungal volvatoxin A2. Cyt2Bb, from Bt ser. *jegathesan* (30.1 kDa) displayed mosquitocidal activity against *A. aegypti* larvae. The toxicity was lower than that of Cyt1Aa; however, the two proteins shared similar haemolytic activity [106]. Cyt2Bc from Bt ser. *medellin* (29.7 kDa) showed mosquitocidal activities against *A. aegypti, An. stephensi, C. pipiens* and *C. quinquefasciatus*. However, the toxicity was lower than Cyt1Aa and Cyt2Ba and trypsin treatment was needed for its haemolytic activity [108]. 

*Dickeya dadantii* plant pathogenic bacteria contain Cyt-like proteins that are active against the pea aphid *Acyrthosiphon pisum* (Hemiptera; Aphididae), but no anti-dipteran activity has been reported [176].

### 4.2. Anti-Dipteran Toxins from Other Microorganisms

#### 4.2.1. Toxins from *Lysinibacillus sphaericus*

The bacterium *L. sphaericus* produces a range of proteins that display toxicity against *C. quinquefasciatus* and other mosquitoes. The Mtx1Aa (formerly Mtx1) protein was first identified in strain SSII-1 [177] and is highly active against *C. quinquefasciatus* and also displays activity against *A. aegypti* larvae and its cells in culture [118]. This protein is a member of the ADP-ribosyl transferase class of toxin and the Mtx1Aa protein (~100 kDa) is cleaved within the mosquito gut to produce an ~70 kDa binding component and the ~27 kDa enzymatic portion of the toxin [118,178]. Mtx1Aa also shows a lower level of activity against *Chironomus riparius* (Diptera; Chironomidae) larvae but no activity against *Drosophila melanogaster* (Diptera; Drosophilidae)*, Simulium* (Diptera; Simuliidae) species or the predatory mosquito *Toxorhynchites splendens* (Diptera; Culicidae) [118,119]. 

The Mpp2 (formerly Mtx2) protein was also identified in *L. sphaericus* SSII-1 [179] and, as a member of the Etx/Mtx2 structural class, is unrelated to the Mtx1Aa protein [179] and is somewhat less toxic to *C. quinquefasciatus* larvae [66]. *L. sphaericus* strains also encode further members of this structural class: Mpp3 (formerly Mtx3) [121] and Mpp4 (formerly Mtx4), in addition to the presence of a further related pseudogene [180]. The activity of Mpp4 has not been tested but Mpp3 is toxic to *C. quinquefasciatus* and weakly active against *A. aegypti* [121]. The relative activity of Mpp2 proteins to these two target insects is different for natural variants of the toxin, and the amino acid residue 224 has been shown to be critical in determining the optimal target; threonine favours activity against *A. aegypti*, whereas lysine favours activity against *C. quinquefasciatus* [120]. 

Highly larvicidal strains of *L. sphaericus* also produce a binary toxin composed of two members of the Toxin_10 structural class, Tpp1Aa (formerly BinA, 42 kDa) and Tpp2Aa (formerly BinB, 51 kDa), where the Tpp2Aa protein is the primary binding component of the toxin and mediates the regional binding and internalization of the Tpp1Aa protein in the *C. quinquefasciatus* midgut [128]. This two component toxin is highly active against *C. quinquefasciatus, C. pipiens, Aedes atropalpus, An. gambiae, An. stephensi,* less active against *An. albimanus,* and *Anopheles quadrimaculatus*, and shows very low to zero activity against *A. aegypti* [126,127]. 

Some strains of *L. sphaericus* also contain another two component toxin named Cry48/Tpp49 (formerly Cry49), [125]. Cry48Aa is a three-domain Cry toxin, and is closely related to the Cry4 toxins, while Tpp49Aa is a member of the Toxin_10 family, like the Tpp1Aa and Tpp2Aa proteins described above. Neither Cry48Aa nor Tpp49Aa were toxic when assayed individually to *C. quinquefasciatus*, but when the proteins were co-administered at the optimum 1:1 ratio, high levels of toxicity against this mosquito species were observed. No activity was detected against other Diptera (*A. aegypti, An. gambiae, Ch. riparius*) or a range of other insects in the orders Coleoptera and Lepidoptera [181]. 

The toxicity of *L. sphaericus* strains to a wide range of mosquito species and to a *Phlebotomus patatasi* (Diptera: Psychodidae) has been reported [182,183] but, since individual toxins were not assessed, the contribution of particular proteins to this activity is unclear. 

#### 4.2.2. Toxins from *Paraclostridium bifermentans*

The toxicity of *P. bifermentans* ser. *malaysia* (formerly *Clostridium bifermentans* ser. *malaysia)* strains to mosquito larvae has been demonstrated [184,185]; however, information on the activity of individual toxins is conflicting. Barloy et al. reported the mosquitocidal activity of the 3-domain protein Cry16Aa1 against *A. aegypti, C. pipiens* and *An. stephensi* [186]. However, a later report tested Cry16Aa1, a combination of Cry16Aa1 co-expressed with another 3-domain protein (Cry17Aa1) and two haemolysin-like proteins (Cbm17.1 and Cbm17.2) from this bacterium and showed no toxicity, alone or in combination, to *C. pipiens, A. aegypti* or *An. gambiae* [187]. A further study also indicated that all four proteins were non-toxic to *A. aegypti* and *An. gambiae* but that when the operon co-expressing all four proteins was used, a high level toxicity to *A. aegypti* was observed [87]. Even with the use of this whole operon, no activity against *An. gambiae* was seen in this work despite the fact that the parental *P. bifermentans* strain has a high toxicity to this mosquito, implying that other factors are involved in this activity. 

Recently, an unrelated protein from *P. bifermentans,* PMP1 (paraclostridial mosquitocidal protein 1) has been described [124]. This clostridial BoNT-like neurotoxin acts through its metalloprotease activity on a neuronal SNARE protein, syntaxin1A. Recombinant PMP1 showed injection toxicity against both larvae and adults of *Anopheles coluzzii* and *A. aegypti*, and to adult *D. melanogaster* (Diptera; Drosophilidae). However, oral toxicity was only observed against *An. coluzzii* and only when PMP1 was co-expressed with one other protein from its operon (NTNH -30% mortality) or with the whole operon including *ntnh* and three *orfX* genes (70% mortality). NTNH may protect the PMP1 protein while the role of the OrfX proteins is not clear [124]. 

#### 4.2.3. Other Dipteran-Active Proteins

Monalysin from *Pseudomonas entomophila* is a pore forming toxin which contributes to the virulence of the bacterium against *Drosophila* (and insects in a range of other orders) by inducing intestinal cell damage [122]. This protein appears to share the fold of aerolysin-like toxins but lacks a putative receptor binding domain. 

Several proteins associated with the canoe-shaped parasporal body of *Brevibacillus laterosporus* are toxins acting against *Musca domestica* (Diptera; Muscidae). Specifically, four highly conserved proteins (ExsC, CHRD, CpbA and CpbB) function as fly virulence factors [123]. 

The Pra and Prb (formerly PirA and PirB) proteins from *Photorhabdus asymbiotica* showed larvicidal activity against both *A. aegypti* and *A. albopictus.* The activity of clones containing the *pra/prb* operon (co-expressing the proteins) was the most toxic compared to Pra alone, Prb alone, or the mixture of Pra plus Prb [129]. Structural analysis of homologs from *Vibrio haemolyticus* that is active against shrimps, indicates that Prb proteins have structural homology to domains 1 and 2 of the 3-domain Cry toxins while Pra proteins have structural homology to domain 3 of the 3-domain Cry toxins [188]. 

## 5. Toxins with Synergistic Activity against Diptera

For a number of insecticidal bacteria, including Bti, the high toxicity of the complete crystal compared to what would be expected, in the event of an additive effect of the toxicity of each of the proteins that compose it, has been attributed to the synergistic activity of its components. This has been studied in more detail in Bti than in any other Bt serovar. 

Combinations of Cry4Aa+Cry4Ba, Cry11Aa+Cry4Aa, and Cry11Aa+Cry4Aa+Cry4Ba have been shown to interact synergistically for a large number of species classified in the mosquito genera *Aedes*, *Anopheles*, and *Culex* [43,55,58,59,61,189]. Moreover, Cry4Ba had synergistic effect with Cry10Aa against *C. pipiens* [64] and with Cry11Aa against *A. aegypti* and *An. albimanus* larvae [58,70]. Although Cyt1A is the least toxic, it is the strongest synergist among the δ-endotoxins against *A. aegypti* [58,74,79,130]. Cyt1A interacts synergistically with Cry11A against *C. quinquefasciatus* [112] and *An. albimanus* [70], and with Cry4Ba against *An. albimanus* [70]. In addition to significantly reducing the lethal concentration, the synergistic effect of Cyt1A also plays an important role in retarding the appearance of resistance to Cry proteins in the case of *C. quinquefasciatus* [190,191,192]. Furthermore, Cyt2Ba, which is present in very low quantities in Bti crystals, has some synergistic effect with Cry4A [62,131] and shows one of the strongest synergistic interactions described so far with Cry10Aa against *A. aegypti* [62]. Another Cyt protein from Bt ser. *darmstadiensis*, Cyt2Aa2, has highly synergistic activity with Cry4Ba from Bti against *A. aegypti* and *C. quinquefasciatus* larvae [69].

**Figure 3:**
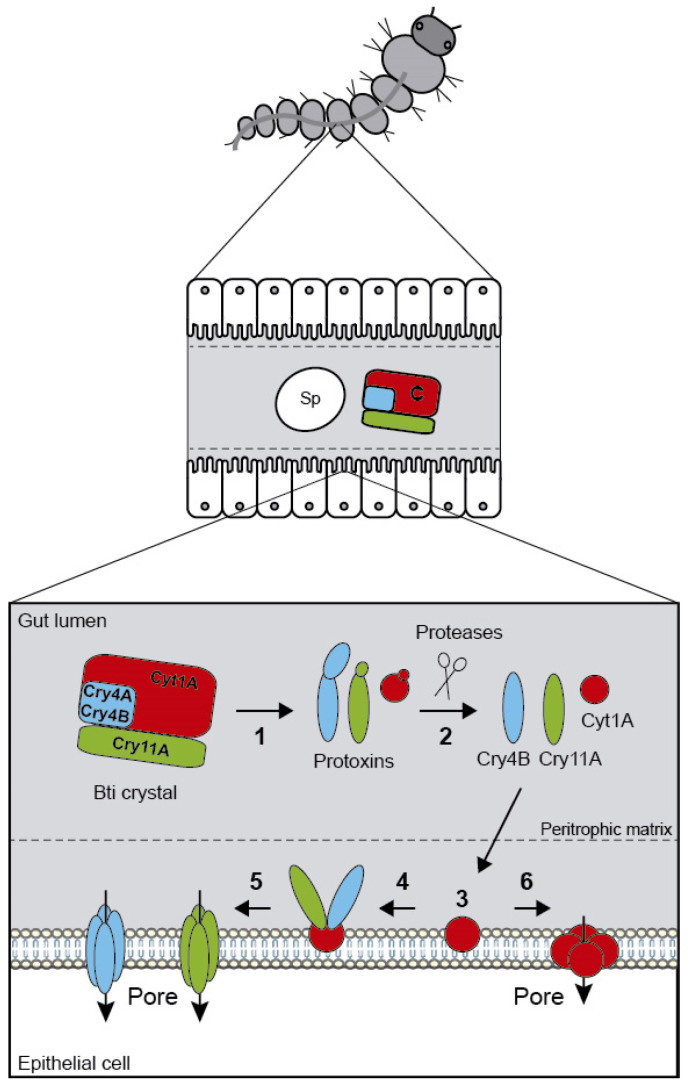
Diagram of the Cyt1Aa synergy mechanism after Bti ingestion by a mosquito larvae. After ingestion of Bti spores and crystals, the crystal is first solubilized (1) and then proteolized and activated by insect gut proteases (2). After traversing the peritrophic matrix, Cyt1Aa protein functions as a membrane-bound receptor for Cry4Ba and Cry11Aa (3 and 4). Finally, Cyt and Cry toxins are able to insert in the membrane to form pores (5 and 6) that lead to osmotic cell lysis. Sp: spore. C: crystal.

The Cyt synergy mechanism has been proposed to be because the Cyt1A protein can function as a membrane-bound receptor for Cry4Ba and Cry11A [193,194]. In relation to Cry11Aa protein, it is suggested that Cyt1Aa inserts its β-sheet into the membrane and two of its components (loop β6-αE and part of β7) bind with high affinity to Cry11Aa, which subsequently is inserted into the larval epithelial membranes [194] (Figure 3). Cyt1Aa seems to facilitate the formation of a pre-pore oligomeric structure that is able to form pores in synthetic lipid membrane vesicles [195,196]. However, oligomerization and membrane insertion of Cyt1A are not essential for its synergistic activity [197]. In the same way, Cyt2Aa2 has been proposed to act as an alternative membrane receptor able to bind specifically to Cry4Ba [198]. 

Synergies have also been described between Cyt proteins and *L. sphaericus* factors and Cyt1Aa, Cyt1Ab and Cyt2Ba combined with *L. sphaericus* binary toxins Tpp1Aa and Tpp2Aa show an increase in toxic activity against *A. aegypti* and *C. quinquefasciatus* [66,107,113,199]. Moreover, Cyt1Aa is also capable of suppressing resistance to *L. sphaericus* toxins in *C. quinquefasciatus* [107,113]. 

Bti Cry toxins can also synergize with proteins from other serovars. For example, Cry11Ba from Bt ser. *jegathesan* and Mpp46Ab (formerly Cry46Ab) from Bt TK-E6 present synergistic activity in combination with Cry4Aa from Bti against *C. pipiens* larvae [65]. Mtx1 and Mpp2 (formerly Mtx2) proteins from *L. sphaericus* were also found to interact synergistically with a variety of Cry-toxins from Bti against *C. quinquefasciatus* [66]. As a result, exploitation of the synergy between different kinds of toxins of Bt or *L. sphaericus* is an excellent strategy to increase the virulence of these microorganisms against relevant dipteran species [84]. 

## 6. Bacterial Insecticides against Mosquitoes

Currently, the major biological control alternatives for mosquito and blackfly larvae are based on bacterial toxins produced by Bti and *L. sphaericus*. Products based on Bti and *L. sphaericus* are marketed and are widely used in the US and Europe, and several different formulations have been developed. The main commercial products are suspension concentrates, followed by wettable powders and, to a lesser extent, large-grained formulations. *L. sphaericus*, because of its better residual activity in polluted waters, has been broadly used against *Culex* species in the US, Central America, Brazil, India, Thailand, and China [200]. For control of mosquito larvae, formulated bacteria are sprayed or spread over the surface of static or slow-moving water into which they sink at a rate determined by the design of the formulation. An innovative formulation of Bti incorporated into ice granules has been used in a mosquito control programme on the Rhine in Germany [201]. Different feeding habits of larvae of different species influence the effectiveness of the bacteria against mosquitoes. *Culex* larvae filter-feed up and down the column of water and they are often termed column feeders, while *Aedes* larvae tend to scavenge along substrate surfaces, particularly on the bottom. *Anopheles* larvae feed on buoyant material trapped at or just below the water surface. In comparable conditions, two *Anopheles* species filtered water at the rate of 33–34 and 49–55 µL/larva/h, respectively, while *C. quinquefasciatus* filtered 490–590 and *A. aegypti* 590–690 µL/larva/h [202]. Larval feeding habits partly explain why species of *Anopheles* have consistently appeared less susceptible to Bt suspensions than the column- and bottom-feeding *Culex* and *Aedes* larvae in laboratory assays and field tests [203]. Thus, differently formulated products are required for mosquito larvae of different feeding types. Buoyant products are required for anophelines, but products should stay in suspension below the surface for column and bottom-feeders. In natural waters, rapid sinking should be avoided because steady deposit of debris would soon cover the particles [204]. Blackfly larvae live in fast-moving water courses and are controlled by pouring bacterial suspensions into the water at consecutive points, from which they are carried downstream [204].

The effectiveness of many bacterial formulations against both mosquitoes and blackflies is short-lived in the field, often only 1–2 days. This is due to rapid settling, adsorption to plants and other substrates (which also filter particles out of the water), denaturing of the crystal by sunlight and engulfment by filter feeding fauna [203,205]. A major goal of the formulation process is to extend the effective period. However, UV radiation inside the water is not as important as particle settling, which is the key factor in determining the effectiveness of a given formulation, since water filters out much of the UV radiation. With Bt, only the effect of sunlight on the crystal reduces larval mortality since the spore is unimportant in mosquito and blackfly larvae. *L. sphaericus* is more susceptible to sunlight, being inactivated in clear water a few centimetres deep in full sun [206], while strong sunlight reduces its effectiveness several-fold [207]. A sunscreen might be beneficial with *L. sphaericus*, particularly in formulations designed to float. 

To increase the effectiveness of active Cry proteins against Diptera, they have been transferred to alternative hosts to increase their persistence in aquatic feeding areas. An improved biopesticide for mosquitoes was developed by inserting *cry* genes from Bti, which is highly toxic to mosquitoes, into the chromosome of *L. sphaericus*, which has longer environmental persistence [208]. The chromosomally integrated *cry* genes were maintained through several generations in the absence of selective pressure. The recombinant *L. sphaericus* producing high levels of *cry11A* gene product from Bti was toxic to *Aedes*, *Culex* and *Anopheles* larvae [209]. A variety of other recombinants have also been produced and tested as reviewed by Federici et al. [84].

## 7. Concluding Remarks and Future Perspectives

The control of dipteran pests is highly relevant as some species in this order are a source of enormous damage in diverse crops, whereas others have potential to transmit serious human diseases.

As the preceding sections show, a number of bacterial species are capable of producing bioinsecticidal proteins that are toxic against this insect order. These bacteria include Bt, the most well-known entomopathogenic bacterium, but also others including *B. laterosporus, L. sphaericus* and *P. bifermentans*. To date, the use of bacteria in the field has been limited to the control of Diptera in the nematoceran suborder and the use of Bt and *L. sphaericus* strains. The use of these bacteria in control programs has often seen great success [210,211]. Specific applications have included control of malaria vectors [212], blackflies as a part of the highly successful onchocerciasis control program [213] and against nuisance mosquitoes in developed countries, for instance in the Upper Rhine, where innovative application techniques were developed [201,214]. Because *L. sphaericus* strains used in the field tend to only produce one spore associated toxin (the binary toxin comprising Tpp1/Tpp2), resistance can arise [215] through mutation of its receptor. Significant resistance to Bti has not been reported in the field and this is likely to be due to its multiple toxins [216] and, particularly, to the role of the Cyt proteins [190,191]. Attempts have been made to combine the activities of Bti and *L. sphaericus* toxins in single strains [217,218] but no recombinant strains have yet been commercialized. Alternative strategies for the deployment of dipteran-active toxins against Nematocera have been explored, including the incorporation of the pesticidal protein genes into a range of other organisms including cyanobacteria [72,219,220], *Caulobacter* [118], gas vacuolated *Ancylobacter* [221] and mosquito gut colonizing *B. cereus* [222], or bioencapsulation in *Tetrahymena* [223] but, again, no product has resulted and the use of naturally-occurring Bt and *L. sphaericus* strains remains the control method of choice.

When considering the other dipteran suborder the Brachycera, as shown above, there are a more limited number of active pesticidal proteins. To the best of the authors’ knowledge, no bacterial pesticides are used specifically for their control and nor have genes encoding pesticidal proteins active against brachyceran insects been incorporated into transgenic plants, targeted at their control. This remains a possibility for the future.

A better understanding of the mode of action for individual proteins against their targets, as well as the molecular interactions occurring between them, including synergism, will help to develop a greater range of tools in the fight against dipteran pests, and help us overcome the appearance of resistance in the future.

## References

[1] Martin P.A.W., Travers R.S. (1989). Worldwide abundance and distribution of *Bacillus thuringiensis* isolates. Appl. Environ. Microbiol..

[2] Iriarte J., Bel Y., Ferrandis M.D., Andrew R., Murillo J., Ferré J., Caballero P. (1998). Environmental distribution and diversity of *Bacillus thuringiensis* in Spain. Syst. Appl. Microbiol..

[3] Jouzani G.S., Valijanian E., Sharafi R. (2017). *Bacillus thuringiensis*: A successful insecticide with new environmental features and tidings. Appl. Microbiol. Biotechnol..

[4] Caballero P., Ferré J., PHYTOMA-España en colaboración con la, Universidad Pública de Navarra (2001). Bioinsecticidas: Fundamentos y Aplicaciones de Bacillus Thuringiensis en el Control Integrado de Plagas.

[5] Federici B.A., Bellows T., Gordh G., Fisher T. (1999). Bacillus thuringiensis. Handbook of Biological Control.

[6] Crickmore N., Berry C., Panneerselvam S., Mishra R., Connor T.R., Bonning B.C. (2020). A structure-based nomenclature for *Bacillus thuringiensis* and other bacteria-derived pesticidal proteins. J. Invertebr. Pathol..

[7] Schnepf E., Crickmore N., Van Rie J., Lereclus D., Baum J., Feitelson J., Zeigler D.R., Dean D.H. (1998). *Bacillus thuringiensis* and its pesticidal crystal proteins. Microbiol. Mol. Biol. Rev..

[8] Wei J.-Z., Hale K., Carta L., Platzer E., Wong C., Fang S.-C., Aroian R. (2003). *Bacillus thuringiensis* crystal proteins that target nematodes. Proc. Natl. Acad. Sci. USA.

[9] Bravo A., Sarjeet G., Mario S. (2007). Mode of action of *Bacillus thuringiensis* Cry and Cyt toxins and their potential for insect control. Natl. Inst. Acess.

[10] Bravo A., Likitvivatanavong S., Gill S.S., Soberon M. (2011). *Bacillus thuringiensis*: A story of a successful bioinsecticide. Insect Biochem. Mol. Biol..

[11] Gonzalez J.J., Dulmage H.T., Carlton B. (1981). Correlation between specific plasmids and delta-endotoxin production in *Bacillus thuringiensis*. Plasmid.

[12] Jurat-Fuentes J.L., Jackson T.A. (2012). Bacterial entomopathogens.

[13] Agaisse H., Lereclus D. (1995). How does *Bacillus thuringiensis* produce so much insecticidal crystal protein?. J. Bacteriol..

[14] De Maagd R.A., Bravo A., Berry C., Crickmore N., Schnepf H.E. (2003). Structure, diversity, and evolution of protein toxins from spore-forming entomopathogenic bacteria. Annu. Rev. Genet..

[15] Xu C., Wang B.C., Yu Z., Sun M. (2014). Structural Insights into *Bacillus thuringiensis* Cry, Cyt and Parasporin Toxins. Toxins.

[16] De Maagd R.A., Bosch D., Stiekema W. (1999). *Bacillus thuringiensis* toxin-mediated insect resistance in plants. Trends Plant Sci..

[17] Bravo A., Gómez I., Porta H., García-Gómez B.I., Rodriguez-Almazan C., Pardo L., Soberón M. (2013). Evolution of *Bacillus thuringiensis* Cry toxins insecticidal activity. Microb. Biotechnol..

[18] De Maagd R.A., Bravo A., Crickmore N. (2001). How *Bacillus thuringiensis* has evolved specific toxins to colonize the insect world. Trends Genet..

[19] Pardo-López L., Soberón M., Bravo A. (2013). *Bacillus thuringiensis* insecticidal three-domain Cry toxins: Mode of action, insect resistance and consequences for crop protection. FEMS Microbiol. Rev..

[20] Berry C., Crickmore N. (2017). Structural classification of insecticidal proteins—Towards an in silico characterisation of novel toxins. J. Invertebr. Pathol..

[21] Butko P. (2003). Cytolytic toxin Cyt1A and its mechanism of membrane damage: Data and hypotheses. Appl. Environ. Microbiol..

[22] Cohen S., Albeck S., Ben-Dov E., Cahan R., Firer M., Zaritsky A., Dym O. (2011). Cyt1Aa toxin: Crystal structure reveals implications for its membrane-perforating function. J. Mol. Biol..

[23] Cohen S., Dym O., Albeck S., Ben-Dov E., Cahan R., Firer M., Zaritsky A. (2008). High-Resolution crystal structure of activated Cyt2Ba monomer from *Bacillus thuringiensis* subsp. *Isr*. J. Mol. Biol..

[24] White I.M., Elson-Harris M.M. (1992). Fruit Flies of Economic Significance: Their Identifiction and Bionomics.

[25] Wheeler M.R., Ashburner M., Thompson J.N., Carson H.L. (1986). Additions to the catalog of the world’s Drosophilidae. The Genetics and Biology of Drosophila.

[26] Vidal-Quist J.C., Castañera P., González-Cabrera J. (2010). Cyt1Aa protein from *Bacillus thuringiensis* (Berliner) serovar *israelensis* is active against the Mediterranean fruit fly, *Ceratitis capitata* (Wiedemann). Pest Manag. Sci..

[27] Leibee G.L. (1984). Influence of temperature on development and fecundity of *Liriomyza trifolii* (Burgess) (Diptera: Agromyzidae) on Celery. Environ. Entomol..

[28] Minkenberg O.P.J.M. (1988). Dispersal of *Liriomyza trifolii*. EPPO Bull..

[29] McPheron B.A., Steck G.J., Taylor & Francis Inc. (1996). Fruit Fly Pests: A World Assessment of Their Biology and Management.

[30] EPPO A2 List EPPO European and Mediterranean Plant Protection Organization, A2 List of pests recommended for regulation as quarantine pests. https://www.eppo.int/ACTIVITIES/plant_quarantine/A2_list.

[31] WHO http://www.who.int/malaria.

[32] CDC https://www.cdc.gov/.

[33] Zhang Q., Hua G., Adang M.J. (2017). Effects and mechanisms of *Bacillus thuringiensis* crystal toxins for mosquito larvae. Insect Sci..

[34] Haider M., Ward E.S., Ellar D.J. (1987). Cloning and heterologous expression of an insecticidal delta-endotoxin gene from *Bacillus thuringiensis* var. *aizawai* ICI toxic to both lepidoptera and diptera. Gene.

[35] Omolo E.O., James M.D., Osir E.O., Thomson J.A. (1997). Cloning and expression of a *Bacillus thuringiensis* (L1-2) gene encoding a crystal protein active against *Glossina morsitans morsitans* and *Chilo partellus*. Curr. Microbiol..

[36] Zhong C., Ellar D.J., Bishop A., Johnson C., Lin S., Hart E.R. (2000). Characterization of a *Bacillus thuringiensis* δ-endotoxin which is toxic to insects in three orders. J. Invertebr. Pathol..

[37] Johnson C., Bishop A.H., Turner C.L. (1998). Isolation and activity of strains of *Bacillus thuringiensis* toxic to larvae of the housefly (Diptera: Muscidae) and tropical blowflies (Diptera: Calliphoridae). J. Invertebr. Pathol..

[38] Heath A.C.G., Wigley P.J., Shoemaker C.B., Chilcott C.N., Broadwell A.H. (2004). Efficacy of native and recombinant Cry1B protein against experimentally induced and naturally acquired ovine myiasis (fly strike) in sheep. J. Econ. Entomol..

[39] Smith G.P., Merrick J.D., Bone E.J., Ellar D.J. (1996). Mosquitocidal activity of the CryIC δ-endotoxin from *Bacillus thuringiensis* subsp. *aizawai*. Appl. Environ. Microbiol..

[40] Abdul-Rauf M., Ellar D.J. (1999). Toxicity and receptor binding properties of a *Bacillus thuringiensis* CryIC toxin active against both Lepidoptera and Diptera. J. Invertebr. Pathol..

[41] Donovan W.P., Dankocsik C., Gilbert M.P. (1988). Molecular characterization of a gene encoding a 72-kilodalton mosquito-toxic crystal protein from *Bacillus thuringiensis* subsp. *israelensis*. J. Bacteriol..

[42] Park H.W., Bideshi D.K., Johnson J.J., Federici B.A. (1999). Differential enhancement of Cry2A versus Cry11A yields in *Bacillus thuringiensis* by use of the cry3A STAB mRNA sequence. FEMS Microbiol. Lett..

[43] Ricoldi M.C., Soares Figueiredo C., Apparecida Desiderio J. (2018). Toxicity of Cry2 proteins from *Bacillus thuringiensis* subsp. *thuringiensis* strain T01-328 against *Aedes aegypti* (Diptera: Culicidae). Toxicology.

[44] Sims S.R. (1997). Host activity spectrum of the CryIIA *Bacillus thuringiensis* subsp. *kurstaki* protein: Effects on Lepidoptera, Diptera, and non-target arthropods. Southwest. Entomol..

[45] Zhang L., Zhao G., Hu X., Liu J., Li M., Batool K., Chen M., Wang J., Xu J., Huang T. (2017). Cry11Aa interacts with the ATP-Binding protein from *Culex quinquefasciatus* to improve the toxicity. J. Agric. Food Chem..

[46] Moar W.J., Trumble J.T., Hice R.H., Backman P.A. (1994). Insecticidal activity of the CryIIA protein from the NRD-12 isolate of *Bacillus thuringiensis* subsp. *kurstaki* expressed in *Escherichia coli* and *Bacillus thuringiensis* and in a leaf-colonizing strain of *Bacillus cereus*. Appl. Environ. Microbiol..

[47] Misra H.S., Hire R.S., Mahajan S.K. (2002). Cloning and characterization of an insecticidal crystal protein gene from *Bacillus thuringiensis* subspecies *kenyae*. J. Genet..

[48] Hire R.S., Makde R.D., Dongre T.K., Souza S.F.D. (2009). Expression, purification and characterization of the Cry2Aa14 toxin from *Bacillus thuringiensis* subsp. *kenyae*. Toxicon.

[49] Mcneil B.C., Dean D.H. (2011). *Bacillus thuringiensis* Cry2Ab is active on Anopheles mosquitoes: Single D block exchanges reveal critical residues involved in activity. FEMS Microbiol. Lett..

[50] Ahmad W., Nicholls C., Ellar D.J. (1989). Cloning and expression of an entomocidal protein gene from *Bacillus thuringiensis* galleriae toxic to both lepidoptera and diptera. FEMS Microbiol. Lett..

[51] Liang H., Liu Y., Zhu J., Guan P., Li S., Wang S., Zheng A., Liu H., Li P. (2011). Characterization of Cry2-type genes of *Bacillus thuringiensis* strains from soil-isolated of sichuan basin, China. Braz. J. Microbiol..

[52] Widner W.R., Whiteley H.R. (1989). Two highly related insecticidal crystal proteins of *Bacillus thuringiensis* subsp. *kurstaki* possess different host range specificities. J. Bacteriol..

[53] Sevim A., Eryüzlü E., Demirba Z., Demir I. (2012). A novel *cry2Ab* gene from the indigenous isolate *Bacillus thuringiensis* subsp. *kurstaki*. J. Microbiol. Biotechnol..

[54] Abdullah M.A.F., Alzate O., Mohammad M., McNall R.J., Adang M.J., Dean D.H. (2003). Introduction of Culex toxicity into *Bacillus thuringiensis* Cry4Ba by protein engineering. Appl. Environ. Microbiol..

[55] Angsuthanasombat C., Crickmore N., Ellar D.J. (1992). Comparison of *Bacillus thuringiensis* subsp. *israelensis* CryIVA and CryIVB cloned toxins reveals synergism in vivo. FEMS Microbiol. Lett..

[56] Angsuthanasombat C., Crickmore N., Ellar D.J. (1991). Cytotoxicity of a cloned *Bacillus thuringiensis* subsp. *israelensis* CryIVB toxin to an *Aedes aegypti* cell line. FEMS Microbiol. Lett..

[57] Beltrão H., Silva-Filha M.H.N.L. (2007). Interaction of *Bacillus thuringiensis* svar. *israelensis* Cry toxins with binding sites from *Aedes aegypti* (Diptera: Culicidae) larvae midgut. FEMS Microbiol. Lett..

[58] Crickmore N., Bone E.J., Williams J.A., Ellar D.J. (1995). Contribution of the individual components of the δ-endotoxin crystal to the mosquitocidal activity of *Bacillus thuringiensis* subsp. *israelensis*. FEMS Microbiol. Lett..

[59] Delecluse A., Poncet S., Klier A., Rapoport G. (1993). Expression of cryIVA and cryIVB genes, independently or in combination, in a crystal-negative strain of *Bacillus thuningiensis* subsp. *israelensis*. Appl. Environ. Microbiol..

[60] Ito T., Ikeya T., Sahara K., Bando H., Asano S.I. (2006). Cloning and expression of two crystal protein genes, cry30Ba1 and cry44Aa1, obtained from a highly mosquitocidal strain, *Bacillus thuringiensis* subsp. *entomocidus* INA288. Appl. Environ. Microbiol..

[61] Poncet S., Delecluse A., Klier A., Rapoport G. (1995). Evaluation of synergistic interactions among CryIVA, CryIVB, and CryIVD toxic components of *B. thuringiensis* subsp. *israelensis* crystals. J. Invertebr. Pathol..

[62] Valtierra-de-Luis D., Villanueva M., Lai L., Williams T., Caballero P. (2020). Potential of Cry10Aa and Cyt2Ba, two minority δ-endotoxins produced by *Bacillus thuringiensis* ser. *israelensis*, for the control of *Aedes aegypti* larvae. Toxins.

[63] Ward E.S., Ridley A.R., Ellar D.J., Todd J.A. (1986). *Bacillus thuringiensis* var. *israelensis* δ-endotoxin. Cloning and expression of the toxin in sporogenic and asporogenic strains of *Bacillus subtilis*. J. Mol. Biol..

[64] Delécluse A., Bourgouin C., Klier A., Rapoport G. (1988). Specificity of action on mosquito larvae of *Bacillus thuringiensis israelensis* toxins encoded by two different genes. MGG Mol. Gen. Genet..

[65] Hayakawa T., Yoneda N., Okada K., Higaki A., Howlader M.T.H., Ide T. (2017). *Bacillus thuringiensis* Cry11Ba works synergistically with Cry4Aa but not with Cry11Aa for toxicity against mosquito *Culex pipiens* (Diptera: Culicidae) larvae. Appl. Entomol. Zool..

[66] Wirth M.C., Berry C., Walton W.E., Federici B.A. (2014). Mtx toxins from *Lysinibacillus sphaericus* enhance mosquitocidal cry-toxin activity and suppress cry-resistance in *Culex quinquefasciatus*. J. Invertebr. Pathol..

[67] Monnerat R., Pereira E., Teles B., Martins E., Praça L., Queiroz P., Soberon M., Bravo A., Ramos F., Soares C.M. (2014). Synergistic activity of *Bacillus thuringiensis* toxins against *Simulium* spp. larvae. J. Invertebr. Pathol..

[68] Hughes P.A., Stevens M.M., Park H.W., Federici B.A., Dennis E.S., Akhurst R. (2005). Response of larval *Chironomus tepperi* (Diptera: Chironomidae) to individual *Bacillus thuringiensis* var. *israelensis* toxins and toxin mixtures. J. Invertebr. Pathol..

[69] Promdonkoy B., Promdonkoy P., Panyim S. (2005). Co-expression of *Bacillus thuringiensis* Cry4Ba and Cyt2Aa2 in *Escherichia coli* revealed high synergism against *Aedes aegypti* and *Culex quinquefasciatus* larvae. FEMS Microbiol. Lett..

[70] Fernandez-Luna M.T., Lanz-mendoza H., Gill S.S., Bravo A., Soberon M., Miranda-rios J. (2010). An α-amylase is a novel receptor for *Bacillus thuringiensis* ssp. *israelensis* Cry4Ba and Cry11Aa toxins in the malaria vector mosquito *Anopheles albimanus* (Diptera: Culicidae). Environ. Microbiol..

[71] Waalwijk C., Dullemans A., Wiegers G., Smits P. (1992). Toxicity of *Bacillus thuringiensis* variety *israelensis* against tipulid larvae. J. Appl. Entomol..

[72] Angsuthanasombat C., Panyim S. (1989). Biosynthesis of 130-kilodalton mosquito larvicide in the cyanobacterium *Agmenellum quadruplicatum* PR-6. Appl. Environ. Microbiol..

[73] Zhu J., Zheng A., Wang S., Liu H., Li P. (2010). Characterization and expression of cry4Cb1 and cry30Ga1 from *Bacillus thuringiensis* strain HS18-1. J. Invertebr. Pathol..

[74] Hernández-Soto A., Del Rincón-Castro M.C., Espinoza A.M., Ibarra J.E. (2009). Parasporal body formation via overexpression of the Cry10Aa toxin of *Bacillus thuringiensis* subsp. *israelensis*, and Cry10Aa-Cyt1Aa synergism. Appl. Environ. Microbiol..

[75] Delecluse A., Rosso M.L., Ragni A. (1995). Cloning and expression of a novel toxin gene from *Bacillus thuringiensis* subsp. *jegathesan* encoding a highly mosquitocidal protein. Appl. Environ. Microbiol..

[76] Florez A.M., Suarez-Barrera M.O., Morales G.M., Rivera K.V., Orduz S., Ochoa R., Guerra D., Muskus C. (2018). Toxic activity, molecular modeling and docking simulations of *Bacillus thuringiensis* Cry11 toxin variants obtained via DNA shuffling. Front. Microbiol..

[77] Orduz S., Realpe M., Arango R., Murillo L.A., Delecluse A. (1998). Sequence of the cry11Bb11 gene from *Bacillus thuringiensis* subsp. *medellin* and toxicity analysis of its encoded protein. Biochim. Biophys. Acta.

[78] Revina L.P., Kostina L.I., Ganushkina L.A., Mikhailova A.L., Zalunin I.A., Chestukhina G.G. (2004). Reconstruction of *Bacillus thuringiensis* ssp. *israelensis* Cry11A endotoxin from fragments corresponding to its N- and C-moieties restores its original biological activity. Biochemistry.

[79] Wu D., Johnson J.J., Federici B.A. (1994). Synergism of mosquitocidal toxicity between CytA and CrylVD proteins using inclusions produced from cloned genes of *Bacillus thuringiensis*. Mol. Microbiol..

[80] Bukhari D.A.A., Shakoori A.R. (2009). Cloning and expression of *Bacillus thuringiensis* cry11 crystal protein gene in *Escherichia coli*. Mol. Biol. Rep..

[81] Feldmann F., Dullemans A., Waalwijk C. (1995). Binding of the CryIVD toxin of *Bacillus thuringiensis* subsp. *israelensis* to larval dipteran midgut proteins. Appl. Environ. Microbiol..

[82] Chang C., Dai S.M., Frutos R., Federici B.A., Gill S.S. (1992). Properties of a 72-kilodalton mosquitocidal protein from *Bacillus thuringiensis* subsp. *morrisoni* PG-14 expressed in *B. thuringiensis* subsp. *kurstaki* by using the shuttle vector pHT3101. Appl. Environ. Microbiol..

[83] Cheong H., Dhesi R.K., Gill S.S. (1997). Marginal cross-resistance to mosquitocidal *Bacillus thuringiensis* strains in Cry11A-resistant larvae: Presence of Cry11A-like toxins in these strains. FEMS Microbiol. Lett..

[84] Federici B.A., Park H.W., Bideshi D.K., Wirth M.C., Johnson J.J. (2003). Recombinant bacteria for mosquito control. J. Exp. Biol..

[85] Juárez-pérez V., Porcar M., Orduz S., Delécluse A. (2003). Cry29A and Cry30A two novel delta-endotoxins isolated from *Bacillus thuringiensis* serovar *medellin*. Syst. Appl. Microbiol..

[86] Restrepo N., Gutierrez D., Patiño M.M., Thiéry I., Delécluse A., Orduz S. (1997). Cloning, expression and toxicity of a mosquitocidal toxin gene of *Bacillus thuringiensis* subsp. *medellin*. Mem. Inst. Oswaldo Cruz.

[87] Qureshi N., Chawla S., Likitvivatanavong S., Lee H.L., Gill S.S. (2014). The cry toxin operon of *Clostridium bifermentans* subsp. *Malaysia* is highly toxic to *Aedes* larval mosquitoes. Appl. Environ. Microbiol..

[88] Rosso M.L., Delécluse A. (1997). Contribution of the 65-kilodalton protein encoded by the cloned gene *cry19A* to the mosquitocidal activity of *Bacillus thuringiensis* subsp. *jegathesan*. Appl. Environ. Microbiol..

[89] Hwang S.H., Saitoh H., Mizuki E., Higuchi K., Ohba M. (1998). A novel class of mosquitocidal δ-endotoxin, Cry19B, encoded by a *Bacillus thuringiensis* serovar *higo* gene. Syst. Appl. Microbiol..

[90] Lee H., Gill S.S. (1997). Molecular cloning and characterization of a novel mosquitocidal protein gene from *Bacillus thuringiensis* subsp. *fukuokaensis*. Appl. Environ. Microbiol..

[91] Berón C.M., Salerno G.L. (2007). Cloning and characterization of a novel crystal protein from a native *Bacillus thuringiensis* isolate highly active against *Aedes aegypti*. Curr. Microbiol..

[92] Saitoh H., Hwang S.H., Park Y.S., Higuchi K., Mizuki E., Ohba M. (2000). Cloning and characterization of a *Bacillus thuringiensis* serovar *higo* gene encoding a novel class of the δ-endotoxin protein, Cry27A, specifically active on the anopheles mosquito. Syst. Appl. Microbiol..

[93] Tan F., Zheng A., Zhu J., Wang L., Li S., Deng Q., Wang S., Li P., Tang X. (2010). Rapid cloning, identification, and application of one novel crystal protein gene *cry30Fa1* from *Bacillus thuringiensis*. FEMS Microbiol. Lett..

[94] Ito T., Bando H., Asano S. (2006). ichiro Activation process of the mosquitocidal δ-endotoxin Cry39A produced by *Bacillus thuringiensis* subsp. *aizawai* BUN1-14 and binding property to *Anopheles stephensi* BBMV. J. Invertebr. Pathol..

[95] Ito T., Sahara K., Bando H., Asano S. (2002). Cloning and expression of novel crystal protein genes cry39A and 39orf2 from *Bacillus thuringiensis* subsp. *aizawai* Bun1-14 encoding mosquitocidal proteins. J. Insect Biotechnol. Sericology.

[96] Hayakawa T., Sakakibara A., Ueda S., Azuma Y., Ide T., Takebe S. (2017). Cry46Ab from *Bacillus thuringiensis* TK-E6 is a new mosquitocidal toxin with aerolysin-type architecture. Insect Biochem. Mol. Biol..

[97] Gough J.M., Kemp D.H., Akhurst R.J., Pearson R.D., Kongsuwan K. (2005). Identification and characterization of proteins from *Bacillus thuringiensis* with high toxic activity against the sheep blowfly, *Lucilia cuprina*. J. Invertebr. Pathol..

[98] Kongsuwan K., Gough J., Kemp D., McDevitt A., Akhurst R. (2005). Characterization of a new *Bacillus thuringiensis* endotoxin, Cry47Aa, from strains that are toxic to the australian sheep blowfly, *Lucilia cuprina*. FEMS Microbiol. Lett..

[99] Zhang W., Yu S., Peng S., Gong J., Qian J., He J., Dai W., Wang R. (2017). Characterization of a novel mosquitocidal toxin of Cry50Ba and its potential synergism with other mosquitocidal toxins. Toxicon.

[100] Tan F., Zhu J., Tang J., Tang X., Wang S., Zheng A., Li P. (2009). Cloning and characterization of two novel crystal protein genes, *cry54Aa1* and *cry30Fa1*, from *Bacillus thuringiensis* strain BtMC28. Curr. Microbiol..

[101] Zhu J., Zheng A.P., Tan F.R., Wang S.Q., Deng Q.M., Li S.C., Wang L.X., Li P. (2010). Characterisation and expression of a novel holotype crystal protein gene, cry56Aa1, from *Bacillus thuringiensis* strain Ywc2-8. Biotechnol. Lett..

[102] Sun Y., Zhao Q., Xia L., Ding X., Hu Q., Federici B.A., Park H.-W. (2013). Identification and characterization of three previously undescribed crystal proteins from *Bacillus thuringiensis* subsp. *jegathesan*. Appl. Environ. Microbiol..

[103] Zhou Y., Wu Z., Zhang J., Wan Y., Jin W., Li Y., Fang X. (2020). Cry80Aa1, a novel *Bacillus thuringiensis* toxin with mosquitocidal activity to *Culex pipiens pallens*. J. Invertebr. Pathol..

[104] Chilcott C.N., Wigley P.J., Broadwell A.H., Park D.J., Ellar D.J. (1998). Activities of *Bacillus thuringiensis* insecticidal crystal proteins Cyt1Aa and Cyt2Aa against three species of sheep blowfly. Appl. Environ. Microbiol..

[105] Thiery I., Delécluse A., Tamayo M.C., Orduz S. (1997). Identification of a gene for Cyt1A-like hemolysin from *Bacillus thuringiensis* subsp. *medellin* and expression in a crystal-negative *B. thuringiensis* strain. Appl. Environ. Microbiol..

[106] Cheong H., Gill S.S. (1997). Cloning and characterization of a cytolytic and mosquitocidal δ- endotoxin from *Bacillus thuringiensis* subsp. *jegathesan*. Appl. Environ. Microbiol..

[107] Wirth M.C., Georghiou G.P., Malik J.I., Abro G.H. (2000). Laboratory selection for resistance to *Bacillus sphaericus* in *Culex quinquefasciatus* (Diptera: Culicidae) from California, USA. J. Med. Entomol..

[108] Juárez-Pérez V., Guerchicoff A., Rubinstein C., Delecluse A. (2002). Characterization of Cyt2Bc toxin from *Bacillus thuringiensis* subsp. *medellin*. Appl. Environ. Microbiol..

[109] Torres-Quintero M.C., Gómez I., Pacheco S., Sánchez J., Flores H., Osuna J., Mendoza G., Soberón M., Bravo A. (2018). Engineering *Bacillus thuringiensis* Cyt1Aa toxin specificity from dipteran to lepidopteran toxicity. Sci. Rep..

[110] Oestergaard J., Ehlers R.U., Martínez-Ramírez A.C., Real M.D. (2007). Binding of Cyt1Aa and Cry11Aa toxins of *Bacillus thuringiensis* serovar *israelensis* to brush border membrane vesicles of *Tipula paludosa* (Diptera: Nematocera) and subsequent pore formation. Appl. Environ. Microbiol..

[111] Koni P.A., Ellar D.J. (1994). Biochemical characterization of *Bacillus thuringiensis* cytolytic d-endotoxins. Microbiology.

[112] Chang C., Yu Y.M., Dai S.M., Law S.K., Gill S.S. (1993). High-level cryIVD and cytA gene expression in *Bacillus thuringiensis* does not require the 20-kilodalton protein, and the coexpressed gene products are synergistic in their toxicity to mosquitoes. Appl. Environ. Microbiol..

[113] Wirth M.C., Delecluse A., Walton W.E. (2001). Cyt1Ab1 and Cyt2Ba1 from *Bacillus thuringiensis* subsp. *medellin* and *B. thuringiensis* subsp. *israelensis* synergize *Bacillus sphaericus* against *Aedes aegypti* and resistant *Culex quinquefasciatus* (Diptera: Culicidae). Appl. Environ. Microbiol..

[114] Payne J., Narva K.E., Uyeda K.A., Stalder C.J., Michaels T.E. (1995). Bacillus thuringiensisisolate PS201T6 toxin. U.S. Patent.

[115] Promdonkoy B., Chewawiwat N., Tanapongpipat S., Luxananil P., Panyim S. (2003). Cloning and characterization of a cytolytic and mosquito larvicidal δ-endotoxin from *Bacillus thuringiensis* subsp. *darmstadiensis*. Curr. Microbiol..

[116] Yu X., Liu T., Sun Z., Guan P., Zhu J., Wang S., Li S., Deng Q., Wang L., Zheng A. (2012). Co-expression and synergism analysis of Vip3Aa29 and Cyt2Aa3 insecticidal proteins from *Bacillus thuringiensis*. Curr. Microbiol..

[117] Nisnevitch M., Cohen S., Ben-Dov E., Zaritsky A., Sofer Y., Cahan R. (2006). Cyt2Ba of *Bacillus thuringiensis israelensis*: Activation by putative endogenous protease. Biochem. Biophys. Res. Commun..

[118] Thanabalu T., Hindley J., Berry C. (1992). Proteolytic processing of the mosquitocidal toxin from *Bacillus sphaericus* SSII-1. J. Bacteriol..

[119] Partridge M.R., Berry C. (2002). Insecticidal activity of the *Bacillus sphaericus* Mtx1 toxin against *Chironomus riparus*. J. Invertebr. Pathol..

[120] Chan S.W., Thanabalu T., Wee B.Y., Porter A.G. (1996). Unusual amino acid determinants of host range in the Mtx2 family of mosquitocidal toxins. Biochemistry.

[121] Liu J.W., Porter A.G., Wee B.Y., Thanabalu T. (1996). New gene from nine *Bacillus sphaericus* strains encoding highly conserved 35.8-kilodalton mosquitocidal toxins. Appl. Environ. Microbiol..

[122] Opota O., Vallet-Gély I., Vincentelli R., Kellenberger C., Iacovache I., Gonzalez M.R., Roussel A., van der Goot F.G., Lemaitre B. (2011). Monalysin, a novel β-pore-forming toxin from the *Drosophila* pathogen *Pseudomonas entomophila*, contributes to host intestinal damage and lethality. PLoS Pathog..

[123] Marche M.G., Mura M.E., Falchi G., Ruiu L. (2017). Spore surface proteins of *Brevibacillus laterosporus* are involved in insect pathogenesis. Sci. Rep..

[124] Contreras E., Masuyer G., Qureshi N., Chawla S., Dhillon H.S., Lee H.L., Chen J., Stenmark P., Gill S.S. (2019). A neurotoxin that specifically targets *Anopheles* mosquitoes. Nat. Commun..

[125] Jones G.W., Nielsen-Leroux C., Yang Y., Yuan Z., Dumas V.F., Monnerat R.G., Berry C. (2007). A new Cry toxin with a unique two-component dependency from *Bacillus sphaericus*. FASEB J..

[126] Berry C., Hindley J., Ehrhardt A.F., Grounds T., De Souza I., Davidson E.W. (1993). Genetic determinants of host ranges of *Bacillus sphaericus* mosquito larvicidal toxins. J. Bacteriol..

[127] Davidson E.W. (1989). Variation in binding of *Bacillus sphaericus* toxin and wheat germ agglutinin to larval midgut cells of six species of mosquitoes. J. Invertebr. Pathol..

[128] Oei C., Hindley J., Berry C. (1992). Binding of purified *Bacillus sphaericus* binary toxin and its deletion derivatives to *Culex quinquefasciatus* gut: Elucidation of functional binding domains. J. Gen. Microbiol..

[129] Ahantarig A., Chantawat N., Waterfield N.R., Ffrench-Constant R., Kittayapong P. (2009). PirAB toxin from Photorhabdus asymbiotica as a larvicide against dengue vectors. Appl. Environ. Microbiol..

[130] González-Villarreal S.E., García-Montelongo M., Ibarra J.E. (2020). Insecticidal activity of a Cry1Ca toxin of *Bacillus thuringiensis* Berliner (Firmicutes: Bacillaceae) and its synergism with the Cyt1Aa toxin against *Aedes aegypti* (Diptera: Culicidae). J. Med. Entomol..

[131] Manasherob R., Itsko M., Sela-Baranes N., Ben-Dov E., Berry C., Cohen S., Zaritsky A. (2006). Cyt1 Ca from *Bacillus thuringiensis* subsp. *israelensis*: Production in *Escherichia coli* and comparison of its biological activities with those of other Cyt-like proteins. Microbiology.

[132] Goldberg L.J., Margalith J. (1977). A bacterial spore demonstrating rapid larvicidal activity against *Anopheles sergentii*, *Uranotaenia unguiculata*, *Culex univitattus*, *Aedes aegypti* and *Culex pipiens*. Mosq. News.

[133] Fillinger U., Knols B.G.J., Becker N. (2003). Efficacy and efficiency of new *Bacillus thuringiensis* var. israelensis and *Bacillus sphaericus* formulations against afrotropical anophelines in western Kenya. Trop. Med. Int. Health.

[134] Margalith Y., Ben-Dov E., Recheigl J.E., Recheigl N.A. (2000). Biological control by *Bacillus thuringiensis* subsp. *israelensis*. Insect Pest Management: Techniques for Environmental Protection.

[135] Ben-Dov E. (2014). *Bacillus thuringiensis* subsp. *israelensis* and its dipteran-specific toxins. Toxins (Basel).

[136] Berry C., Ben-dov E., Jones A.F., Murphy L., Quail M.A., Holden M.T.G., Harris D., Zaritsky A., Parkhill J. (2002). Complete sequence and organization of pBtoxis, the toxin-coding plasmid of *Bacillus thuringiensis* subsp. *israelensis*. Appl. Environ. Microbiol..

[137] Bolotin A., Gillis A., Sanchis V., Nielsen-LeRoux C., Mahillon J., Lereclus D., Sorokin A. (2017). Comparative genomics of extrachromosomal elements in *Bacillus thuringiensis* subsp. *israelensis*. Res. Microbiol..

[138] Gillis A., Fayad N., Makart L., Bolotin A., Sorokin A., Kallassy M., Mahillon J. (2018). Role of plasmid plasticity and mobile genetic elements in the entomopathogen *Bacillus thuringiensis* serovar *israelensis*. FEMS Microbiol. Rev..

[139] Caballero J., Jiménez-Moreno N., Orera I., Williams T., Fernández A.B., Villanueva M., Ferré J., Caballero P., Ancín-Azpilicueta C. (2020). Unraveling the composition of insecticidal crystal proteins in *Bacillus thuringiensis*: A proteomics approach. Appl. Environ. Microbiol..

[140] Bietlot H.P.L., Vishnubhatla I., Carey P.R., Pozsgay M., Kaplan H. (1990). Characterization of the cysteine residues and disulphide linkages in the protein crystal of *Bacillus thuringiensis*. Biochem. J..

[141] Couche G.A., Pfannenstiel M.A., Nickerson K.W. (1987). Structural disulfide bonds in the *Bacillus thuringiensis* subsp. *israelensis* protein crystal. J. Bacteriol..

[142] Bourgouin C., Delecluse A., Ribier J., Klier A., Rapoport G. (1988). A *Bacillus thuringiensis* subsp. *israelensis* gene encoding a 125-kilodalton larvicidal polypeptide is associated with inverted repeat sequences. J. Bacteriol..

[143] Otieno-Ayayo Z.N., Zaritsky A., Wirth M.C., Manasherob R., Khasdan V., Cahan R., Ben-Dov E. (2008). Variations in the mosquito larvicidal activities of toxins from *Bacillus thuringiensis* ssp. *israelensis*. Environ. Microbiol..

[144] Thorne L., Garduno F., Thompson T., Decker D., Zounes M., Wild M., Waldfield A., Pollock T.J. (1986). Structural similarity between the Lepidoptera- and Diptera-specific insecticidal endotoxin genes of *Bacillus thuringiensis* subsp. *“kurstaki”* and *“israelensis”*. J. Bacteriol..

[145] Lee S.G., Eckblad W., Lee A.B. (1985). Diversity of protein inclusion bodies and identification of mosquitocidal protein in *Bacillus thuringiensis* subsp. *israelensis*. Biochem. Biophys. Res. Commun..

[146] Garduno F., Thorne L., Walfield A.M., Pollock T.J. (1988). Structural relatedness between mosquitocidal endotoxins of *Bacillus thuringiensis* subsp. *israelensis*. Appl. Environ. Microbiol..

[147] Gutierrez P., Alzate O., Orduz S. (2001). A theoretical model of the tridimensional structure of *Bacillus thuringiensis* subsp. *medellin* Cry 11Bb toxin deduced by homology modelling. Mem. Inst. Oswaldo Cruz.

[148] Dervyn E., Poncet S., Klier A., Rapoport G. (1995). Transcriptional regulation of the cryIVD gene operon from *Bacillus thuringiensis* subsp. *israelensis*. J. Bacteriol..

[149] Wu D., Federici B.A. (1995). Improved production of the insecticidal CryIVD protein in *Bacillus thuringiensis* using cryIA(c) promoters to express the gene for an associated 20 kDa protein. Appl. Microbiol. Biotechnol..

[150] Yamagiwa M., Sakagawa K., Sakai H. (2004). Functional analysis of two processed fragments of *Bacillus thuringiensis* Cry11A toxin. Biosci. Biotechnol. Biochem..

[151] Dai S.M., Gill S.S. (1993). In vitro and in vivo proteolysis of the *Bacillus thuringiensis* subsp. *israelensis* CryIVD protein by *Culex quinquefasciatus* larval midgut proteases. Insect Biochem. Mol. Biol..

[152] Fernandez L.E., Aimanova K.G., Gill S.S., Bravo A., Soberón M. (2006). A GPI-anchored alkaline phosphatase is a functional midgut receptor of Cry11Aa toxin in *Aedes aegypti* larvae. Biochem. J..

[153] Chen J., Aimanova K.G., Pan S., Gill S.S. (2009). Identification and characterization of *Aedes aegypti* aminopeptidase N as a putative receptor of *Bacillus thuringiensis* Cry11A toxin. Insect Biochem. Mol. Biol..

[154] Chen J., Aimanova K.G., Fernandez L.E., Bravo A., Soberon M., Gill S.S. (2009). *Aedes aegypti* cadherin serves as a putative receptor of the Cry11Aa toxin from *Bacillus thuringiensis* subsp. *israelensis*. Biochem. J..

[155] Pérez C., Fernández L.E., Gill S.S., Segovia L., Bravo A., Rodríguez M.H., Soberón M. (2005). Cry11Aa toxin from *Bacillus thuringiensis* binds its receptor in *Aedes aegypti* mosquito larvae through loop α-8 of domain II. FEBS Lett..

[156] Likitvivatanavong S., Gill S.S., Bravo A., Soberón M. (2011). Cadherin, alkaline phosphatase, and aminopeptidase N as receptors of Cry11Ba toxin from *Bacillus thuringiensis* subsp. *jegathesan* in *Aedes aegypti*. Appl. Environ. Microbiol..

[157] Anderson I., Sorokin A., Kapatral V., Reznik G., Bhattacharya A., Mikhailova N., Burd H., Joukov V., Kaznadzey D., Walunas T. (2005). Comparative genome analysis of *Bacillus cereus* group genomes with *Bacillus subtilis*. FEMS Microbiol. Lett..

[158] Chilcott C.N., Wigley P.J. (1994). Opportunities for finding new *Bacillus thuringiensis* strains. Agric. Ecosyst. Environ..

[159] Saleem F., Shakoori A.R. (2010). Characterization of cry2A-type gene(s) from pakistani isolates of *Bacillus thuringiensis* toxic to lepidopteran and dipteran insects. Pak. J. Zool..

[160] Nicholls C.N., Ahmad W., Ellar D.J. (1989). Evidence for two different types of insecticidal P2 toxins with dual specificity in *Bacillus thuringiensis* subspecies. J. Bacteriol..

[161] Goje L.J., Elmi E.D., Bracuti A., Courty T., Rao T., Alzahrani F.A., Crickmore N. (2020). Identification of *Aedes aegypti* specificity motifs in the N-terminus of the *Bacillus thuringiensis* Cry2Aa pesticidal protein. J. Invertebr. Pathol..

[162] Shu C., Zhang F., Chen G., Joseph L., Barqawi A., Evans J., Song F., Li G., Zhang J., Crickmore N. (2017). A natural hybrid of a *Bacillus thuringiensis* Cry2A toxin implicates Domain I in specificity determination. J. Invertebr. Pathol..

[163] Chilcott C., Ellar D. (1988). Comparative toxicity of *Bacillus thuringiensis* var. *israelensis* crystal proteins in vivo and in vitro. J. Gen. Microbiol..

[164] Adams L.F., Visick J.E., Whiteley H.R. (1989). A 20-kilodalton protein is required for efficient production of the *Bacillus thuringiensis* subsp. *israelensis* 27-kilodalton crystal protein in *Escherichia coli*. J. Bacteriol..

[165] Al-yahyaee S.A.S., Ellar D.J. (1995). Maximal toxicity of cloned CytA δ-endotoxin from *Bacillus thuringiensis* subsp. *israelensis* requires proteolytic processing from both the N- and C-termini. Microbiology.

[166] Gill S.S., Singh G.J.P., Hornung J.M. (1987). Cell membrane interaction of *Bacillus thuringiensis* subsp. *israelensis* cytolytic toxins. Infect. Immun..

[167] Haider M.Z., Ellar D.J. (1989). Mechanism of action of *Bacillus thuringiensis* insecticidal δ-endotoxin: Interaction with phospholipid vesicles. BBA Biomembr..

[168] Knowles B.H., Blatt M.R., Tester M., Horsnell J.M., Carroll J., Menestrina G., Ellar D.J. (1989). A cytolytic δ-endotoxin from *Bacillus thuringiensis* var. *israelensis* forms cation-selective channels in planar lipid bilayers. FEBS Lett..

[169] Butko P., Huang F., Pusztai-Carey M., Surewicz W.K. (1996). Membrane permeabilization induced by cytolytic δ-endotoxin CytA from *Bacillus thuringiensis* var. *israelensis*. Biochemistry.

[170] Butko P., Huang F., Pusztai-Carey M., Surewicz W.K. (1997). Interaction of the δ-endotoxin CytA from *Bacillus thuringiensis* var. *israelensis* with lipid membranes. Biochemistry.

[171] Anaya P., Onofre J., Torres-Quintero M.C., Sánchez J., Gill S.S., Bravo A., Soberón M. (2020). Oligomerization is a key step for *Bacillus thuringiensis* Cyt1Aa insecticidal activity but not for toxicity against red blood cells. Insect Biochem. Mol. Biol..

[172] Orduz S., Diaz T., Restrepo N., Patiño M.M., Tamayo M.C. (1996). Biochemical, immunological and toxicological characteristics of the crystal proteins of *Bacillus thuringiensis* subsp. *medellin*. Mem. Inst. Oswaldo Cruz.

[173] Valtierra-de-Luis D., Villanueva M., Caballero J., Matas I.M., Williams T., Caballero P. (2019). Quantification of dose-mortality responses in adult Diptera: Validation using *Ceratitis capitata* and *Drosophila suzukii* responses to spinosad. PLoS ONE.

[174] Guerchicoff A., Ugalde R.A., Rubinstein C.P. (1997). Identification and characterization of a previously undescribed cyt gene in *Bacillus thuringiensis* subsp. *israelensis*. Appl. Environ. Microbiol..

[175] Li J., Koni P.A., Ellar D.J. (1996). Structure of the mosquitocidal δ-endotoxin CytB from *Bacillus thuringiensis* sp. *kyushuensis* and implications for membrane pore formation. J. Mol. Biol..

[176] Loth K., Costechareyre D., Effantin G., Rahbé Y., Condemine G., Landon C., Da Silva P. (2015). New Cyt-like δ -endotoxins from *Dickeya dadantii*: Structure and aphicidal activity. Sci. Rep..

[177] Thanabalu T., Hindley J., Jackson-Yap J., Berry C. (1991). Cloning, sequencing, and expression of a gene encoding a 100-kilodalton mosquitocidal toxin from *Bacillus sphaericus* SSII-1. J. Bacteriol..

[178] Thanabalu T., Berry C., Hindley J. (1993). Cytotoxicity and ADP-ribosylating activity of the mosquitocidal toxin from *Bacillus sphaericus* SSII-1: Possible roles of the 27- and 70-kilodalton peptides. J. Bacteriol..

[179] Thanabalu T., Porter A.G. (1996). A *Bacillus sphaericus* gene encoding a novel type of mosquitocidal toxin of 31.8 kDa. Gene.

[180] Berry C. (2012). The bacterium, *Lysinibacillus sphaericus*, as an insect pathogen. J. Invertebr. Pathol..

[181] Jones G.W., Wirth M.C., Monnerat R.G., Berry C. (2008). The Cry48Aa-Cry49Aa binary toxin from *Bacillus sphaericus* exhibits highly restricted target specificity. Environ. Microbiol..

[182] Silva Filha M.H.N.L., Berry C., Regis L. (2014). Lysinibacillus sphaericus: Toxins and mode of action, applications for mosquito control and resistance management. Advances in Insect Physiology.

[183] Pener H., Wilamowski A. (1996). Susceptibility of larvae of the sandfly *Phlebotomus papatasi* (Diptera: Psychodidae) to *Bacillus sphaericus*. Bull. Entomol. Res..

[184] Yiallouros M., Storch V., Thiery I., Becker N. (1994). Efficacy of *Clostridium bifermentans* serovar *Malaysia* on target and nontarget organisms. J. Am. Mosq. Control Assoc..

[185] Nicolas L., Charles J.-F., de Barjac H. (1993). *Clostridium bifermentans* serovar *malaysia*: Characterization of putative mosquito larvicidal proteins. FEMS Microbiol. Lett..

[186] Barloy F., Delécluse A., Nicolas L., Lecadet M.M. (1996). Cloning and expression of the first anaerobic toxin gene from *Clostridium bifermentans* subsp. *malaysia*, encoding a new mosquitocidal protein with homologies to *Bacillus thuringiensis* delta-endotoxins. J. Bacteriol..

[187] Juárez-Pérez V., Delécluse A. (2001). The cry toxins and the putative hemolysins of *Clostridium bifermentans* ser. *malaysia* are not involved in mosquitocidal activity. J. Invertebr. Pathol..

[188] Lee C.T., Chen I.T., Yang Y.T., Ko T.P., Huang Y.T., Huang J.Y., Huang M.F., Lin S.J., Chen C.Y., Lin S.S. (2015). The opportunistic marine pathogen *Vibrio parahaemolyticus* becomes virulent by acquiring a plasmid that expresses a deadly toxin. Proc. Natl. Acad. Sci. USA.

[189] Soberón M., López-Díaz J.A., Bravo A. (2013). Cyt toxins produced by *Bacillus thuringiensis*: A protein fold conserved in several pathogenic microorganisms. Peptides.

[190] Georghiou G.P., Wirth M.C. (1997). Influence of exposure to single versus multiple toxins of *Bacillus thuringiensis* subsp. *israelensis* on development of resistance in the mosquito *Culex quinquefasciatus* (Diptera: Culicidae). Appl. Environ. Microbiol..

[191] Wirth M.C., Georghiou G.P. (1997). Cross-Resistance among CryIV toxins of *Bacillus thuringiensis* subsp *israelensis* in *Culex quinquefasciatus* (Diptera: Culicidae). J. Econ. Entomol..

[192] Wirth M.C., Park H.W., Walton W.E., Federici B.A. (2005). Cyt1A of *Bacillus thuringiensis* delays evolution of resistance to Cry11A in the mosquito *Culex quinquefasciatus*. Appl. Environ. Microbiol..

[193] Cantón P.E., Reyes E.Z., Ruiz De Escudero I., Bravo A., Soberon M. (2011). Binding of *Bacillus thuringiensis* subsp. *israelensis* Cry4Ba to Cyt1Aa has an important role in synergism. Peptides.

[194] Perez C., Fernandez L.E., Sun J., Folch J.L., Gill S.S., Soberon M., Bravo A. (2005). *Bacillus thuringiensis* subsp. *israelensis* Cyt1Aa synergizes Cry11Aa toxin by functioning as a membrane-bound receptor. Proc. Natl. Acad. Sci. USA.

[195] Pérez C., Muñoz-Garay C., Portugal L.C., Sánchez J., Gill S.S., Soberón M., Bravo A. (2007). *Bacillus thuringiensis* ssp. *israelensis* Cyt1Aa enhances activity of Cry11Aa toxin by facilitating the formation of a pre-pore oligomeric structure. Cell. Microbiol..

[196] Elleuch J., Tounsi S., Belguith Ben Hassen N., Lacoix M.N., Chandre F., Jaoua S., Zribi Zghal R. (2015). Characterisation of novel *Bacillus thuringiensis* isolates against *Aedes aegypti* (Diptera: Culicidae) and *Ceratitis capitata* (Diptera: Tephridae). J. Invertebr. Pathol..

[197] López-Diaz J.A., Cantón P.E., Gill S.S., Soberón M., Bravo A. (2013). Oligomerization is a key step in Cyt1Aa membrane insertion and toxicity but not necessary to synergize Cry11Aa toxicity in *Aedes aegypti* larvae. Environ. Microbiol..

[198] Lailak C., Khaokhiew T., Promptmas C., Promdonkoy B., Pootanakit K., Angsuthanasombat C. (2013). *Bacillus thuringiensis* Cry4Ba toxin employs two receptor-binding loops for synergistic interactions with Cyt2Aa2. Biochem. Biophys. Res. Commun..

[199] Thiéry I., Hamon S., Delécluse A., Orduz S. (1998). The introduction into *Bacillus sphaericus* of the *Bacillus thuringiensis* subsp. medellin *cyt1Ab1* gene results in higher susceptibility of resistant mosquito larvae populations to *B. sphaericus*. Appl. Environ. Microbiol..

[200] Wirth M.C. (2010). Mosquito resistance to bacterial larvicidal toxins. Open Toxinology J..

[201] Becker N. (2003). Ice granules containing endotoxins of microbial agents for the control of mosquito larvae—A new application technique. J. Am. Mosq. Control Assoc..

[202] Aly C. (1988). Filtration rates of mosquito larvae in suspensions of latex microspheres and yeast cells. Entomol. Exp. Appl..

[203] Lacey L.A., Smittle B.J. (1985). The effects of gamma radiation on espore viability and mosquito larvicidal activity of *Bacillus sphaericus* and *Bacillus thuringiensis* var. *israelensis*. Bull. Soc. Vector Ecol..

[204] Burges H.D. (1998). Formulation of Microbial Biopesticides: Beneficial Microorganisms, Nematodes and Seed Treatments.

[205] Lacey L. (1986). Microbial control of black flies and mosquitoes. Annu. Rev. Entomol..

[206] Mulligan F.S., Schaefer C.H., Wilder W.H. (1980). Efficacy and persistence of *Bacillus sphaericus* and *B. thuringiensis* H. 14 against mosquitoes under laboratory and field conditions. J. Econ. Entomol..

[207] Skovmand O., Bauduin S. (1998). Efficacy of a granular formulation of *Bacillus sphaericus* against *Culex quinquefasciatus* and *Anopheles gambiae* in west african countries. J. Vector Ecol..

[208] Bar E., Sandler N., Makayoto M., Keynan A. (1998). Expression of chromosomally inserted *Bacillus thuringiensis israelensis* toxin genes in *Bacillus sphaericus*. J. Invertebr. Pathol..

[209] Poncet S., Bernard C., Dervyn E., Cayley J., Klier A., Rapoport G. (1997). Improvement of *Bacillus sphaericus* toxicity against dipteran larvae by integration, via homologous recombination, of the Cry11A toxin gene from *Bacillus thuringiensis* subsp. *israelensis*. Appl. Environ. Microbiol..

[210] Lacey L.A. (2007). Bacillus thuringiensis serovariety israelensis and Bacillus sphaericus for mosquito control. J. Am. Mosq. Control Assoc..

[211] Priest F.G. (1992). Biological control of mosquitoes and other biting flies by *Bacillus sphaericus* and *Bacillus thuringiensis*. J. Appl. Bacteriol..

[212] Ingabire C.M., Hakizimana E., Rulisa A., Kateera F., Van Den Borne B., Muvunyi C.M., Mutesa L., Van Vugt M., Koenraadt C.J.M., Takken W. (2017). Community-based biological control of malaria mosquitoes using *Bacillus thuringiensis* var. *israelensis* (Bti) in Rwanda: Community awareness, acceptance and participation. Malar. J..

[213] Lacey L.A., Escaffre H., Philippon B., Sékétéli A., Guillet P. (1982). Large river treatment with *Bacillus thuringiensis* (H-14) for the control of *Simulium damnosum* s.l. in the onchocerciasis control programme. Trop. Parasitol.

[214] Becker N. (1997). Microbial control of mosquitoes: Management of the upper rhine mosquito population as a model programme. Parasitol. Today.

[215] Chalegre K.D.D.M., Romão T.P., Amorim L.B., Anastacio D.B., De Barros R.A., De Oliveira C.M.F., Regis L., De-Melo-Neto O.P., Silva-Filha M.H.N.L. (2009). Detection of an allele conferring resistance to *Bacillus sphaericus* binary toxin in *Culex quinquefasciatus* populations by molecular screening. Appl. Environ. Microbiol..

[216] Bonin A., Paris M., Frérot H., Bianco E., Tetreau G., Després L. (2015). The genetic architecture of a complex trait: Resistance to multiple toxins produced by *Bacillus thuringiensis israelensis* in the dengue and yellow fever vector, the mosquito *Aedes aegypti*. Infect. Genet. Evol..

[217] Gammon K., Jones G.W., Hope S.J., De Oliveira C.M.F., Regis L., Silva Filha M.H.N.L., Dancer B.N., Berry C. (2006). Conjugal transfer of a toxin-coding megaplasmid from *Bacillus thuringiensis* subsp. *israelensis* to mosquitocidal strains of *Bacillus sphaericus*. Appl. Environ. Microbiol..

[218] Federici B.A., Park H., Bideshi D.K. (2010). Overview of the basic biology of *Bacillus thuringiensis* with emphasis on genetic engineering of bacterial larvicides for mosquito control. Open Toxinology J..

[219] Csiszàr K., Houmard J., Damerval T., de Marsac N.T. (1987). Transcriptional analysis of the cyanobacterial gvpABC operon in differentiated cells: Occurrence of an antisense RNA complementary to three overlapping transcripts. Gene.

[220] Xu X., Kong R., Hu Y. (1993). High larvicidal activity of intact recombinant cyanobacterium *Anabaena* sp. PCC 7120 expressing Gene 51 and Gene 42 of *Bacillus sphaericus* sp. 2297. FEMS Microbiol. Lett..

[221] Wai H.Y., Thanabalu T., Porter A.G. (1994). Expression of mosquitocidal toxin genes in a gas-vacuolated strain of *Ancylobacter aquaticus*. Appl. Environ. Microbiol..

[222] Luxananil P., Tanapongpipat S., Promdonkoy B., Atomi H., Imanaka T., Panyim S. (2003). Expression of binary toxin genes in the mosquito-colonizable bacteria, *Bacillus cereus*, leads to high toxicity against *Culex quinquefasciatus* larvae. Curr. Microbiol..

[223] Zaritsky A., Zalkinder V., Ben-Dov E., Barak Z. (1991). Bioencapsulation and delivery to mosquito larvae of *Bacillus thuringiensis* H14 toxicity by *Tetrahymena pyriformis*. J. Invertebr. Pathol..

